# Identification of RNA helicases with unwinding activity on angiogenin-processed tRNAs

**DOI:** 10.1093/nar/gkad033

**Published:** 2023-01-31

**Authors:** Aleksej Drino, Lisa König, Charlotte Capitanchik, Nasim Sanadgol, Eva Janisiw, Tom Rappol, Elisa Vilardo, Matthias R Schaefer

**Affiliations:** Division of Cell and Developmental Biology, Center for Anatomy and Cell Biology, Medical University of Vienna, Schwarzspanierstr. 17-I, A-1090 Vienna, Austria; Division of Cell and Developmental Biology, Center for Anatomy and Cell Biology, Medical University of Vienna, Schwarzspanierstr. 17-I, A-1090 Vienna, Austria; RNA Networks Laboratory, Francis Crick Institute, London, UK; Division of Cell and Developmental Biology, Center for Anatomy and Cell Biology, Medical University of Vienna, Schwarzspanierstr. 17-I, A-1090 Vienna, Austria; Division of Cell and Developmental Biology, Center for Anatomy and Cell Biology, Medical University of Vienna, Schwarzspanierstr. 17-I, A-1090 Vienna, Austria; Division of Cell and Developmental Biology, Center for Anatomy and Cell Biology, Medical University of Vienna, Schwarzspanierstr. 17-I, A-1090 Vienna, Austria; Division of Cell and Developmental Biology, Center for Anatomy and Cell Biology, Medical University of Vienna, Schwarzspanierstr. 17-I, A-1090 Vienna, Austria; Division of Cell and Developmental Biology, Center for Anatomy and Cell Biology, Medical University of Vienna, Schwarzspanierstr. 17-I, A-1090 Vienna, Austria

## Abstract

Stress-induced tRNA fragmentation upon environmental insult is a conserved cellular process catalysed by endonucleolytic activities targeting mature tRNAs. The resulting tRNA-derived small RNAs (tsRNAs) have been implicated in various biological processes that impact cell-to-cell signalling, cell survival as well as gene expression regulation during embryonic development. However, how endonuclease-targeted tRNAs give rise to individual and potentially biologically active tsRNAs remains poorly understood. Here, we report on the *in vivo* identification of proteins associated with stress-induced tsRNAs-containing protein complexes, which, together with a ‘tracer tRNA’ assay, were used to uncover enzymatic activities that can bind and process specific endonuclease-targeted tRNAs *in vitro*. Among those, we identified conserved ATP-dependent RNA helicases which can robustly separate tRNAs with endonuclease-mediated ‘nicks’ in their anticodon loops. These findings shed light on the existence of cellular pathways dedicated to producing individual tsRNAs after stress-induced tRNA hydrolysis, which adds to our understanding as to how tRNA fragmentation and the resulting tsRNAs might exert physiological impact.

## INTRODUCTION

Besides their canonical function during protein translation, tRNAs represent a source of a heterogeneous class of small RNAs called tRNA-derived small RNAs (tsRNAs). Currently, tsRNAs are classified according to their parental tRNA isoacceptor identity, position of tRNA cleavage events as well as their length (1). Specific tsRNA species, namely 5′ and 3′ tRNA halves, have been repeatedly detected during cellular responses to stress such as starvation (2), oxidation, hypoxia and hypothermia (3,4) as well as after heat shock or irradiation (5). The RNase A-family member Angiogenin (ANG) is the main nuclease in mammals responsible for fragmenting tRNAs during the stress response (3,6). It does so by targeting pyrimidine-purine dinucleotides in single-stranded regions of tRNAs, with preference for the anticodon (AC)-loop (7). Various tsRNA identities, including those that resulted also from hydrolysis events in D-, variable and T-loops) have been implicated in numerous biological processes, including proliferation, differentiation and translation (8–13), control of transposon activity (14), intercellular communication (15,16), regulation of vertebrate embryogenesis (17) and transfer of extra-chromosomal information between generations (18,19). However, apart from a few exceptions (11,13,20–22), how exactly individual tsRNAs act at the molecular level remains largely unclear.

Besides the existence of redundant (stress-induced) anticodon nucleases creating tRNA halves (23–25), and the likely existence of other RNases targeting the D- and T-loops, little is known about the mechanistic details of tsRNA biogenesis after tRNA hydrolysis. Most notably, a major unresolved question relates to the molecular steps following tRNA hydrolysis by endonucleases. Since tRNAs are highly structured molecules, formed by numerous secondary and tertiary interactions, which are further reinforced by post-transcriptional nucleotide modifications (26), hydrolysis events in any tRNA loop structure will not destabilise such ‘nicked tRNAs’ to an extent that would result in the complete separation of 5′ from 3′ tsRNAs. However, most reports have attributed biological impact to individual 5′ and 3′ tsRNAs (reviewed in (27)). Furthermore, many reports on tsRNAs abundance, including small RNA sequencing experiments after stress exposure, injury or ANG overexpression, revealed an asymmetric recovery of 5' and 3' tsRNAs from cells with particular ‘jackpot’ tsRNAs such as 5' tsRNA-Gly^GCC/CCC^ and 5' tsRNA-Glu^CUC/UUC^ as overrepresented in various data sets (21,23,28,29). While biases in cDNA library preparation resulting from RNA modifications could be responsible for these observations (30), experimental manipulation of biological systems with specific sequences mimicking 5' tsRNAs (5,8,11,12,21,28,31) or 3' tsRNAs (13,32–35) suggested impact of individual tsRNA moieties on specific cellular processes. If this holds true, this would require the existence of cellular activities with the ability to separate 5′ from 3′ tsRNAs after endonucleolytic attack on parental tRNAs. Yet, the identity of such activities is currently unknown.

Here, we combined the biochemical enrichment and identification of tsRNAs-containing ribonucleoprotein particles (RNPs) from stressed cells with a versatile *in vitro* activity assay, which allows characterizing cellular activities that can act on ‘nicked tRNAs’ *in vitro*. Using these approaches, we identified various conserved ATP-dependent RNA helicases that can separate ANG-hydrolysed tRNAs. For one of them, DDX3X, we dissected tRNA substrate preference as well as tRNA binding and unwinding kinetics towards ANG-hydrolysed tRNAs and report that DDX3X function affects the stability of 3′ tsRNAs *in vivo*. These findings implicate multiple RNA helicases in the biogenesis of individual tsRNAs and pave the way for further elucidating the molecular details of tsRNA biogenesis as a prerequisite for understanding the biological function of these small RNAs.

## MATERIALS AND METHODS

### Tissue culture

HEK293, U2OS, HeLa cells and murine bone marrow-derived fibroblasts (BMDF) were cultured in Dulbecco's modified Eagle's medium (DMEM, Thermo) supplemented with 10% FBS (v/v), 2 mM l-glutamine, 100 U/ml penicillin and 100 μg/ml streptomycin (DMEM-F/G/P/S). HAP1 cells were cultivated in IMDM medium supplemented with 10% fetal calf serum. Cells were maintained in a humidified incubator at 37°C and 5% CO_2_ and split when 70–80% confluent using trypsin–EDTA (0.05%, Thermo).

### RNA helicase knock-down using siRNAs in cell culture

HeLa cells were seeded in 6-well plates and transfected with control siRNAs or siRNAs targeting DDX3X or/and DDX5 (see oligonucleotide sequences) at a final concentration of 25 nM using DharmaFECT1 transfection reagent (Horizon Discovery) following the manufacturer's recommendations. After 72 h, cells were collected for cytoplasmic protein extract preparation or RNA extraction.

### DDX3X knock-down by short-hairpin RNAs

HeLa or HEK293 cells were infected with 1:20 dilution of lentivirus preparations (produced by using a third-generation lentiviral system in HEK293T cells) expressing a shRNA construct targeting human DDX3X (cloned into pLKO.1, see oligonucleotide sequences) in DMEM-F/G/P/S supplemented with 10 μg/ml of polybrene transfection reagent (Sigma). After 24 h of infection, cells were transferred to selection media containing 300 μg/ml of G418 for 72 h prior to experimental manipulation.

### DDX3X depletion by cre-mediated recombination

Mouse BMDF containing a ‘floxed’ DDX3X allele were incubated in DMEM-F/G/P/S supplemented with 500 nM 4-hydroxy-tamoxifen (4-OHT, Sigma) for at least 72 h before experimental manipulation.

### Rescue-of-function of DDX3X by transient transfection of pCMV-mDDX3X-HA

Mouse BMDF were seeded in 57 cm^2^ dishes, and different plasmid masses encoding a CMV-driven HA-tagged murine DDX3X gene were transfected using poly-ethylenimine (PEI, Sigma), following the manufacturer's recommendations. After 24 h, BMDF were incubated in medium supplemented with 500 nM 4-hydroxy-tamoxifen (4-OHT, Sigma) for 72 h before protein extraction or experimental manipulation.

### Stress paradigm

For induction of tRNA fragmentation, cultured cells were incubated in media supplemented with 0.5 mM inorganic sodium meta-arsenite (As[III], Sigma) prior to experimental manipulation (2 h for size exclusion, or 1 h for stress granule core enrichment, or 1 h for northern blotting of total RNA).

### tRNA isolation under denaturing conditions

Cells were washed once in 1× PBS, scraped off the culture plate in 1 ml 1× PBS, centrifuged for 2 min at 500 g at 4°C and then resuspended in 200 μl 1× PBS. 1 ml of Trizol (Sigma) was immediately added followed by incubation for 10 min on ice. After adding 200 μl of chloroform, samples were centrifuged at 21 000 g for 10 min at room temperature. The aqueous phase was re-extracted with an equal volume of chloroform, followed by centrifugation at 21 000 g for 5 min at room temperature. The aqueous phase was precipitated using an equal volume of isopropanol and 1 μl GlycoBlue (Ambion, 15 mg/ml) for ≧ 15 min at room temperature. RNA was collected by centrifugation at 21 000 g for 30 min at 4°C. RNA pellets were washed once using 75% ethanol, followed by air-drying ≦ 5 min at room temperature. Total RNAs extracted from cultured cells were separated by urea-PAGE (6%) using 0.5× TBE, followed by staining of RNA using SYBR-Gold (Invitrogen). RNAs were visualised using a gel documentation system (Biorad), tRNAs were excised from PA gel and eluted in 3 volumes of gel extraction buffer (300 mM sodium acetate; 1 mM EDTA; 0.1% SDS). Eluted tRNAs were precipitated using an equal volume of isopropanol and 1 μl GlycoBlue (15 mg/ml) for ≧ 15 min at room temperature. tRNAs were resuspended in water and the concentration was determined by NanoDrop (ThermoScientific).

### tRNA isolation under native conditions

Cells were washed once in 1× PBS supplemented with 5 mM MgCl_2_ (PBS-Mg), scraped off the culture plate into 1 ml PBS-Mg, centrifuged for 2 min at 500 g at 4°C and resuspended in 500 μl of PBS–Mg. 500 μl of acidic phenol (Roth) was immediately added followed by incubation for 10 min on a rotating wheel. Samples were centrifuged at 16 000 g for 7 min at room temperature. The aqueous phase was re-extracted with an equal volume of chloroform, followed by centrifugation at 21 000 g for 5 min at room temperature. The aqueous phase was precipitated using an equal volume of isopropanol and 1 μl GlycoBlue (15 mg/ml) for ≧ 15 min at room temperature. RNA was collected by centrifugation at 21 000 g for 30 min at 4°C. RNA pellets were washed once using 75% ethanol/5mM MgCl_2_, followed by air-drying ≦ 5 min at room temperature. Total RNAs extracted from cultured cells under native conditions were separated by native PAGE (6%) using 0.5× TB supplemented with 5 mM MgCl_2_, followed by staining of RNA using SYBR-Gold (Invitrogen). RNAs were visualised using a gel documentation system (Biorad), tRNAs were excised from PA gel and eluted in 3 volumes of gel extraction buffer containing 5 mM MgCl_2_. Eluted tRNAs were precipitated using an equal volume of isopropanol and 1 μl GlycoBlue (15 mg/ml) for ≧ 15 min at room temperature. Eluted tRNAs were resuspended in 10 mM MgCl_2_ and the concentration was determined by NanoDrop (ThermoScientific).

### Purification of specific tRNA isoacceptors by RNA affinity capture

Total RNA extracted from 5 × 145 cm^2^ dishes (HeLa cells) was re-suspended in 10 ml of IEX-buffer A (20 mM Tris; 10 mM KCl; 1.5 mM MgCl_2_). For ion exchange chromatography, a HiTrap Q FF anion exchange chromatography column (1 ml, Cytiva) was used on an ÄKTA-FPLC (Cytiva) at 4°C and RNA was eluted in IEX-buffer B (20 mM Tris; 1 M NaCl; 10 mM KCl; 1.5 mM MgCl_2_). Small RNAs eluting between 400–600 mM NaCl were collected and precipitated using isopropanol at –20°C. The small RNA pool was resuspended in water, melted by incubation at 75°C for 3 min, immediately placed on ice and 20 ml NHS-binding buffer (30 mM HEPES–KOH; 1.2 M NaCl; 10 mM MgCl_2_) was added. For RNA affinity capture, 80 μg of a 5' amino-modified DNA oligonucleotide complementary to the target tRNA (IDT) was covalently coupled to a HiTrap^TM^ NHS-activated HP column (1 ml, Cytiva). tRNA binding to the NHS-column was performed by recirculation of the small RNA pool for 4 h minimum at 60°C and thereafter washed using NHS-washing buffer (2.5 mM HEPES–KOH; 0.1 M NaCl; 10 mM MgCl_2_). Bound RNAs were eluted by submerging the column in a water bath at 75°C in NHS elution buffer (0.5 mM HEPES–KOH; 1 mM EDTA) followed by immediate precipitation in isopropanol at –20°C. Precipitated RNA was re-suspended in water. Purified tRNA-Gly^GCC/CCC^ or tRNA-Lys^UUU/CUU^ was further gel-purified (8% urea–PAGE in 0.5× TBE) using RNA gel extraction buffer (0.3 M sodium acetate, pH 5.2; 0.1% SDS; 1 mM EDTA) and immediately precipitated in isopropanol at –20°C.

### Preparation of 5′ end-labelled tRNAs

Total tRNAs were extracted from cultured cells by urea-PAGE (6%) as described above. One hundred (100) nanograms of extracted full-length tRNAs (4 pmol) or affinity-purified tRNAs (tRNA-Gly^GCC/CCC^, tRNA-Lys^UUU/CUU^) were de-phosphorylated using alkaline phosphatase (1 unit of FastAP, Thermo) for 20 min at 37°C, followed by re-extraction with acidic phenol/chloroform/isoamyl alcohol (P/C/I, 25:24:1, Roth) and isopropanol precipitation. tRNAs were resuspended in water and 5′ end-labelled using T4 PNK and ^32^P-γ-ATP at 37°C for 60 min. Unincorporated ^32^P-γ-ATP was removed by desalting using spin columns (P-6DG polyacrylamide, BioRad). 5′ end-labelled tRNAs were precipitated in 0.3 M sodium acetate (pH 5.2) and 1 μl GlycoBlue (15 mg/ml) in an equal volume of isopropanol for 18 h at 4°C. tRNA was collected by centrifugation at 21 000 g for 30 min at 4°C. tRNA pellets were washed once using 75% ethanol, followed by air-drying ≦ 5 min at room temperature. tRNAs were resuspended in water and stored in aliquots at –80°C until use.

### Preparation of 3′ end-labelled tRNAs

Total tRNAs were extracted from cultured cells by urea-PAGE (6%) as described above. One hundred nanograms of extracted tRNA (about 4 pmol) were labelled using T4 RNA ligase 1 (NEB) and 5'-^32^P-cytidine 3',5'-bisphosphate (pCp) at 37°C for 60 min. Unincorporated pCp was removed by desalting using spin columns (P-6DG polyacrylamide, BioRad). 3′ end-labelled tRNAs were precipitated in 0.3 M sodium acetate (pH 5.2) and 1 μl GlycoBlue (15 mg/ml) in an equal volume of isopropanol for 18 h at 4°C. tRNA was collected by centrifugation at 21 000 g for 30 min at 4°C. RNA pellets were washed once using 75% ethanol, followed by air-drying ≦ 5 min at room temperature. tRNAs were resuspended in water, melted at 75°C for 3 min and immediately placed on ice. T4 PNK buffer A was added to 1× (final), followed by incubation for 10 min at room temperature. Re-folded tRNAs were processed for ANG-mediated hydrolysis as described for 3‘-tracer tRNAs.

### Preparation of ‘tracer tRNAs’ using recombinant ANG

For 3′-tracer tRNAs, 100 ng (4 pmol) of native or refolded tRNA or specific affinity-purified tRNA were treated with 1 μl of recombinant ANG (rANG, R&D, stock concentration 6 μM in 1× PBS containing 1 mg/l BSA) in T4 PNK buffer A at 37°C for 30 min with intermittent mixing by pipetting. Spermine (Sigma) was added to a final concentration of 5 μM, and the reaction was incubated at 37°C for an additional 30 min. After rANG-mediated tRNA hydrolysis, ‘nicked’ tRNA duplexes were labelled using T4 PNK and ^32^P-γ-ATP at 37°C for 60 min. 3′-tracer tRNAs were re-precipitated in 1× T4 PNK Buffer A supplemented with sodium acetate (pH 5.2) to a final concentration of 0.3 M final, 1 μl GlycoBlue (15 mg/ml) in an equal volume of isopropanol for 18 h at 4°C. 3′-tracer tRNAs were used for either RNA helicase activity assays or EMSA.

For 5′-tracer tRNAs, 5′ labelled tRNAs (see above) were resuspended in water, melted at 75°C for 3 min and immediately placed on ice. T4 PNK buffer A (1 μl) was added, followed by incubation for 10 min at room temperature. Re-folded tRNAs were incubated with rANG as described for 3′-tracer tRNAs. 5′-tracer tRNAs were used for either RNA helicase activity assays or EMSA.

### Northern blotting (NB)

RNA was separated by 12% urea–PAGE, followed by RNA transfer to Hybond nylon membranes (Cytiva) in 0.5× TBE using a semi-dry blotting device for 30 min at 10 V = const. Blotted RNA was immobilised by UV-crosslinking with 1200 mJ/cm^2^ (Stratalinker) followed by baking of membranes at 60°C for 18 h. Complementary DNA oligonucleotides to tRNA sequences were radio-labelled using T4 PNK and ^32^P-γ-ATP. Unincorporated ^32^P-γ-ATP was removed by desalting using spin columns (P-6DG polyacrylamide, BioRad). Hybridization was performed at 40°C for ≧ 4 h in a hybridization buffer (5× SSC, 20 mM Na_2_HPO_4_ pH 7.4, 7% SDS, 1× Denhardt's reagent). Membranes were washed once for 10 min with wash buffer A (3× SSC, 5% SDS) at 40°C, followed by wash buffer B (1× SSC, 1% SDS) for 10 min at room temperature. Membranes were exposed to storage phosphor screens (Cytiva) and imaged using a Typhoon imager (Cytiva).

### Cytoplasmic protein extraction

Cells were collected using trypsin–EDTA (0.05%, Thermo), washed with 1× PBS, and incubated in a hypotonic buffer (30 mM HEPES–KOH, pH 7.4; 100 mM potassium acetate; 2 mM Mg(OAc)_2_; 2 mM NaF; 5 mM DTT, 0.1% NP-40) supplemented with murine RNase inhibitor (5 U/100 μl (NEB)) and protease inhibitors (Roche) for 10 min on ice. Cells were broken by passage (20×) through a hypodermic needle (26 G). Intact cells were discarded after centrifugation at 300 g for 7 min at 4°C, followed by removal of nuclei through centrifugation at 1000 g for 10 min at 4°C. The concentration of the cytoplasmic protein extracts was measured using Bradford reagent (Biorad), followed by snap-freezing aliquots (10 μg) in liquid nitrogen and storage at -80°C until use.

### Preparation of HEK293 cells for biochemical fractionation

5 × 145 cm^2^ dishes (HEK293 cells) at 80% confluency were acutely exposed to As[III] or left untreated (controls), followed by 2 washes using 10 ml of ice-cold 1× PBS. Cells were covered with a thin film of ice-cold 1× PBS and exposed to UV light (254 nm) with 400 mJ/cm^2^ (Stratagene). Cells were scraped off the culture plate into 15 ml 1× PBS, centrifuged for 3 min at 500 g at 4°C. Cell pellets were flash-frozen in liquid nitrogen and stored at –80°C until further processing.

### Chromatographic fractionation of whole protein lysates and tracing of tRNAs

1 ml of ice-cold SEC lysis buffer (20 mM Tris, pH 7.4; 150 mM NaCl; 10 mM MgCl_2_; 10% glycerol; 1 mM DTT; 0.2% NP-40) supplemented with 1× protease inhibitors (Roche) was added to one cell pellet (obtained from 5 × 145 cm^2^ dishesculture dishes) and cells were slowly thawed on ice. Cells were lysed by passage (20x) through a hypodermic needle (26 G), followed by centrifugation at 21 000 x g for 5 min at 4°C. Soluble protein was saved, followed by re-extraction of the cell pellet as described above. The resulting (pooled) soluble protein extract (2 ml) was filtered (45 μm) and loaded onto a HiLoad® 26/600 Superdex® 200 pg SEC column pre-equilibrated in column running buffer (20 mM Tris, pH 7.4; 150 mM NaCl; 1 mM DTT) at 4°C. Gel filtration was performed on a ÄKTA Purifier FPLC system (Cytiva) for the duration of 1.1 column volume (CV) at a flow rate of 2.5 ml/min. Fractions (2 ml) were collected with a delay of 0.25 CV to avoid fractionation prior to void volume peak elution. 750 μl of each fraction was incubated with proteinase K (final concentration of 2 mg/ml) at 37°C for 10 min under constant mixing (thermo-mixer). RNAs were extracted by adding an equal volume of acidic P/C/I and precipitated using an equal volume of isopropanol and 1 μl GlycoBlue (15 mg/ml). RNA pellets were analysed by NB using DNA oligonucleotides for specific tRNAs. 5′ tsRNAs-containing SEC fractions were thawed on ice, pooled and subjected to anion exchange chromatography (IEX) at 4°C on a ÄKTA Purifier FPLC system (Cytiva) using HiTrap Q Fast Flow columns (Cytiva, 1 ml) with a flow rate of 1 ml/min in IEX buffer A (20 mM Tris, pH 7.4; 1.5 mM MgCl_2_; 10 mM KCl). A linear gradient (40–60%) of IEX buffer B (20 mM Tris, pH 7.4; 1.5 mM MgCl_2_; 10 mM KCl and 1M NaCl) was applied for the duration of 20 CV while fractions (750 μl) were collected. Subsequently, RNA was extracted from 250 μl of each IEX fraction followed by NB for specific tRNAs.

### LC–MS/MS on 5′ tsRNAs-containing RNPs

IEX fractions exhibiting a positive 5′ tsRNA-Gly^GCC/CCC^/tRNA-Gly^GCC/CCC^ signal ratio on NB were concentrated to an approximate volume of 50 μl using a SpeedVac Vacuum Concentrator on high temperature settings (60°C). 12.5 μl of 4× SDS-sample buffer was added to each fraction, followed by denaturation for 2 min at 90°C. Fractions were separated by modified SDS-PAGE prepared by overlaying equal volumes of stacking (4%) and resolving (6%) gels. Pre-stained protein marker (Thermo) was used to monitor sample migration through the stacking gel. Once samples reached the resolving gel, migration was allowed for approximately one centimetre, followed by fixation and staining of gels using the Colloidal Blue staining kit (Invitrogen). Excised protein samples were processed for in-gel tryptic digestion and analysis by LC–MS/MS at the Max Perutz Laboratories (MPL) mass spectrometry facility. Specifically, gel sections were excised, cut up, and de-stained with acetonitrile (ACN) and 50 mM ammonium bicarbonate (ABC). Before each of the following reaction steps, gel pieces were washed with 50 mM ABC in 50% ACN and dehydrated in 100% ACN in order to facilitate the uptake of the solutions. Disulfide bridges were reduced in 10 mM dithiothreitol in 50 mM ABC for 30 min at 56°C. Subsequently, free thiols were alkylated with 50 mM iodoacetamide in 50 mM ABC in the dark (30 min at RT). Proteins were digested with trypsin (Promega) in 50 mM ABC overnight at 37°C. The reaction was stopped by adding 10 μl of 10% (v/v) formic acid (FA) and peptides were extracted by sonication with 5% FA. Digests were concentrated and desalted using custom-made C18 stage-tips. The purified peptides were separated on an Ultimate 3000 RSLC nano-flow chromatography system (Thermo Fisher Scientific), using a pre-column for sample loading (PepMapAcclaim C18, 2 cm × 0.1 mm, 5 μm) and a C18 analytical column (PepMapAcclaim C18, 50 cm × 0.75 mm, 2 μm, both Dionex-Thermo Fisher Scientific), applying a linear gradient from 2 to 35% solvent B (B: 80% acetonitrile, 0.08% FA; solvent A: 0.1% FA) at a flow rate of 230 nl/min over 60 min. Eluting peptides were analysed on a Q Exactive HF Orbitrap mass spectrometer equipped with a Proxeon nanospray source (Thermo Fisher Scientific), operated in data-dependent mode. Survey scans were recorded in a scan range of 375–1500 *m*/z, at a resolution of 60 000 at 200 *m*/*z* and an AGC target value of 3E6. The eight most intense ions were selected with an isolation width of 1.6 Da, fragmented in the HCD cell at 27% collision energy and the spectra recorded at a target value of 1E5 and a resolution of 30 000. Peptides with a charge of +1 or >+6 were excluded from fragmentation, the peptide match and exclude isotope features were enabled and selected precursors were dynamically excluded from repeated sampling for 15 s. MaxQuant software package, version 1.6.0.16 was used to identify the proteins searching against the human uniprot database (2018.07_UP000005640_9606_Homo_sapiens_all) plus the sequences of common contaminants with tryptic specificity allowing two missed cleavages. Oxidised methionine, acetylation of the protein N-terminus was set as variable, carbamidomethylation of cysteines as fixed modification. Results were filtered at a false discovery rate of 1% at the protein and PSM level.

### LC–MS/MS data analysis

Following computational MaxQuant analysis of individual LC–MS/MS runs, data was analysed using the following steps: (i) protein intensities of each sample (consisting of 4 fractions) were combined resulting in three values originating from control or As[III] exposure experiments (sum of protein intensities), respectively; (ii) since samples were prepared, processed and measured at different days, and to off-set experimental variation such as protein concentration or machine performance, the sum of protein intensities was normalised within each experimental group (control and As[III] exposure) using the mean intensity of a set of three cytoskeletal proteins (sp|P60709|ACTB_HUMAN Actin, sp|P68363|TBA1B_HUMAN Tubulin α-1B chain, sp|P68371|TBB4B_HUMAN Tubulin β-4B chain); (iii) resulting normalised protein intensities were log_2_-transformed; (iv) any missing values were separately imputed using a normal distribution for each of the six intensity columns (three originating from control or As[III] exposure experiments, respectively); (v) The mean intensity of the values originating from As[III] exposure versus control experiments were used as a measure of protein enrichment, including only those identities with at least two valid values in one of the experimental groups.

### Enrichment of SG cores and co-immunoprecipitation from SG cores

HeLa cells were grown to 80% confluency, exposed to As[III] (0.5 mM for 60 min) and collected by trypsinization. Cells were washed once in 1× PBS, pelleted and flash-frozen in liquid nitrogen. SG core enrichment was performed as described in (36). Final SG-enriched fractions were subjected to co-immunoprecipitation using antibodies (4 μg) against eIF4A1 (Abcam, ab31217) or DDX3X (Bethyl Labs, A300-475A) coupled to 30 μl (pre-cleared) Dynabeads™ Protein A (Thermo). Immunoprecipitations were performed for 2 h at 4°C. Aliquots were taken at representative steps of the enrichment protocol, protein concentrations were determined using Bradford reagent, and equal mass of total proteins along with the content of the co-immunoprecipitations were analysed by western blotting.

### Western blotting

For total protein extracts, collected cells were lysed in RIPA buffer (10 mM Tris–HCl, pH 8.0; 1 mM EDTA; 0.5 mM EGTA; 1% Triton X-100; 0.1% SDS; 140 mM NaCl), followed by incubation for 10 min on ice and centrifugation for 10 min at 21 000 g at 4°C. Protein extracts or aliquots of concentrated IEX fractions were mixed with SDS sample buffer to 1×, denatured at 95°C for 3 min, followed by separation using SDS-PAGE (12%). Proteins were transferred to a nitrocellulose membrane (Roche) by wet blotting (Biorad) for 1 h at 100 V = const. in transfer buffer (20 mM sodium borate, pH 8.8; 20% methanol; 1 mM DTT; 0.5 mM EDTA). Membranes were blocked for 1 h in western blotting block solution (WB-BS: 5% low-fat dried milk powder in 1× PBS) followed by incubation for 18 h at 4°C with primary antibodies against eIF2α-P (1:500, Abcam ab32157), β-actin (1:5.000, Sigma A5316), vinculin (1:1000, Cell Signalling E1E9V), DDX3X (1:1.000, Abcam ab128206), DDX1 (1:1.000, Bethyl Laboratories A300-521A), DDX6 (1:1000, Abcam ab234837), eIF4A1 (1:1000, Abcam ab31217), DDX5 (1:1000, Bethyl Laboratories A300-523A), DDX17, (1:1000, Abcam ab24601), DDX39 (1:1.000, Abcam ab176348), DHX36 (1:1000, ThermoFisher Scientific PA5-57259), G3BP1 (1:2000, Abcam ab56574), hnRNPA1B2 (1:5000, Abcam ab6102) in WB-BS. Membranes were washed three times for 5 min in WB-BS, followed by incubation for 30 min at room temperature with HRP-coupled secondary antibodies (1: 5000, Jackson Labs) in WBBS. After washing three times for 5 min in 1× PBS and once in 1× PBS/0.1% Tween-20, ECL reagent (BioRad) was added and chemiluminescent signals were collected using a Fusion FX imager (Vilber).

### Indirect immunofluorescence experiments

Cells were seeded in Nunc™ Lab-Tek™ II Chamber Slide™ Systems (8 wells). At a confluency of 70%, cells were either left untreated or were exposed to As[III] (0.3 mM) for 1 h. Cells were rinsed once with 1× PBS and fixed either in 4% paraformaldehyde (PFA) in 1× PBS for 10 min at room temperature, or in 100% ice-cold methanol for 10 min. Fixed cells were washed three times with 1× PBS followed by permeabilization in 1× PBS/0.1% Triton X-100 for 15 min at room temperature. Cells were incubated in immunofluorescence block solution (IF-BS: 3% BSA, 0.1% Triton X-100, 0.05% NaN_3_, 1× PBS) for 30 min at room temperature. Cells were incubated for 18 h in a wet chamber at 4°C with primary antibodies diluted in IF-BS against G3BP1 (1:1000, Abcam ab56574), ANG (1:200, Merck MABC166, clone 26–2F), ANG (1:200, Abcam ab214931), DDX3X (1:200, Abcam ab128206), DDX1 (1:200, Bethyl Laboratories A300-521A), DDX6 (1:500, Abcam ab234837), eIF4A1 (1:500, Abcam ab31217), DDX5 (1:200, Bethyl Laboratories A300-523A), DDX17 (1:200, Abcam ab24601), DDX39 (1:200, Abcam ab176348), DHX36 (1:200, Thermo PA5-57259), DHX9 (1:500, Thermo PA5-19542), DDX21 (1:500, Abcam ab182156). Cells were washed twice for 20 min in IF-BS followed by incubation for 2 h at room temperature in a dilution of secondary fluorophore-coupled antibodies (1:500; Jackson Laboratories) in IF-BS. Cells were washed twice for 20 min in 1× PBS/0.1% Triton X-100, incubated for 5 min in 1× PBS containing 1 μg/ml Hoechst 33342, washed once for 5 min in 1× PBS/0.1% Triton X-100 and mounted on microscopic slides using a drop of ROTI®Mount FluorCare (Roth). Imaging was performed on a laser scanning microscope LSM FV3000 (Olympus).

### Expression and purification of recombinant human DDX3X

The pETM41 expression vectors harbouring a hexa-histidine and MBP (maltose-binding protein) N-terminally fused to human DDX3X or DDX3X_DQAD_ (37) were expressed in *E. coli* BL21(DE3) pLysS (Invitrogen) in LB medium supplemented with kanamycin (50 μg/ml) and chloramphenicol (50 μg/ml). Protein expression was induced at OD_600_ = 0.6 using IPTG (1 mM) for 3 h at 37°C. Bacteria were harvested by centrifugation at 4°C (15 min at 3500 g). Cell pellets were resuspended in 20 ml His-Buffer A (50 mM Tris, pH 8; 10 mM imidazole; 500 mM KCl) supplemented with protease inhibitors (Roche). Cell disruption was performed by sonication on ice (5 cycles, 90 s work/30 s pause). Lysates were cleared by centrifugation for 10 min using 21 000 g at 4°C. Lysates were loaded onto Ni-NTA His Trap High Performance columns (5 ml, Cytiva) that were pre-equilibrated in His-Buffer A. Columns were washed with Buffer A (10 CV) followed by elution of recombinant protein in a 0–100% gradient of His Buffer B (50 mM Tris, pH 8; 500 mM KCl; 1 M imidazole). The elution profile of His-MBP-DDX3X was evaluated using 10% SDS-PAGE and Coomassie-blue staining. Peak fractions (2 ml) containing His-MBP-DDX3X were subjected to gel filtration using a Superdex® 200 10/300 column pre-equilibrated in His-MBP-DDX3X storage buffer (22 mM Tris, pH 8; 330 mM KCl; 1.1 mM DTT) at a 0.5 ml/min flow rate for the duration equivalent to 1.1 CV. All purification steps were performed on the ÄKTA Purifier FPLC system (Cytiva). The elution profile of His-MBP-DDX3X (750 μl fractions) was monitored by SDS-PAGE and Coomassie-blue staining. His-MBP-DDX3X-containing fractions were supplemented with glycerol (final concentration of 10%), aliquots (20 μl) were flash-frozen in liquid nitrogen and stored at –80°C until usage.

### Expression and purification of recombinant murine eIF4A1

The pET3b expression vector harbouring Glutathione S-transferase (GST) C-terminally fused to human eIF4A1 (eIF4A1-GST) (38) was expressed in *E. coli* BL21(DE3) pLysS (Invitrogen) as described above for DDX3X, except for 3 h at 30°C. Cell pellets were resuspended in 10 ml of GST-Buffer A (20 mM Tris, pH 8; 500 mM NaCl; 1 mM DTT; 5% glycerol) supplemented with protease inhibitors (Roche). Cell disruption and clearing of lysate by centrifugation was performed as described for DDX3X. Lysates were loaded onto a GSTrap HiTrap Fast Flow (1 ml, Cytiva) pre-equilibrated in GST-Buffer A. The column was washed with Buffer A (10 CV), followed by elution of recombinant protein in a 0–100% gradient of GST-Buffer B (GST-Buffer A supplemented with 10 mM reduced glutathione). The elution profile of GST-eIF4A1 was evaluated by 10% SDS-PAGE and Coomassie-blue staining. Peak fractions (2 ml) containing eIF4A1-GST were subjected to gel filtration on the ÄKTA Purifier FPLC system (Cytiva) using a Superdex® 200 10/300 column pre-equilibrated in storage buffer (22 mM Tris, pH 8; 165 mM NaCl; 1.1 mM DTT) at a 0.5 ml/min flow rate for the duration equivalent to 1.1 CV. The elution profile of eIF4A1-GST (750 μl fractions) was monitored by SDS-PAGE and Coomassie-blue staining. eIF4A1-GST-containing fractions were supplemented with glycerol (final concentration of 10%), aliquots (25 μl) were flash-frozen in liquid nitrogen and stored at –80°C until usage.

### Expression and purification of recombinant human DDX5

MBP-DDX5-GST was expressed from a pMAL vector harbouring human DDX5 N-terminally fused to MBP and C-terminally fused to GST (39). MBP-DDX5-GST was expressed in *E. coli* BL21(DE3) pLysS (Invitrogen) after induction with 0.2 mM IPTG for 18 h at 16°C, and purified as described for eIF4A1-GST.

### Expression and purification of recombinant human DDX1

The pDEST17 expression vector harbouring DDX1 was transformed into *E. coli* BL21(DE3) pLysS (Invitrogen) and cells were grown in LB medium supplemented with ampicillin (50 μg/ml) and chloramphenicol (50 μg/ml). Protein expression was induced at OD600 = 0.6 using IPTG (1 mM) for 3 h at 37°C. Cells were harvested by centrifugation at 4°C (15 min at 3500 g). Cell pellets were resuspended in 25 ml His-Buffer A (20 mM Tris, pH 8; 500 mM NaCl; 10 mM imidazole; 2 mM DTT; 10% glycerol) supplemented with protease inhibitors (Roche). Cell disruption was performed by sonication on ice (5 cycles, 90 s work/30 s pause). Lysates were cleared by centrifugation for 10 min at 21 000 g and 4°C. Cleared lysates were loaded onto Ni-NTA His Trap High Performance columns (5 ml, Cytiva) that were pre-equilibrated in His-Buffer A. Columns were washed with Buffer A (10 CV) followed by elution of recombinant protein in a 0–100% gradient of His Buffer B (20 mM Tris, pH 8; 500 mM NaCl; 2 mM DTT; 1 M imidazole; 10% glycerol). The elution profile of His-DDX1 was evaluated using 10% SDS-PAGE and Coomassie-blue staining. Peak fractions (2 ml) were diluted in 20 ml ion exchange (IEX) buffer A (50 mM Tris, pH 8; 5 mM MgCl_2_; 2 mM DTT) and loaded on a QFF Column (1 ml, Cytiva) that was pre-equilibrated in IEX Buffer A. Column was washed with Buffer A (10 CV) followed by elution of recombinant protein in IEX Q FF Buffer B (50 mM Tris, pH 8; 5 mM MgCl_2_; 2 mM DTT; 1 M KCl). The elution profile of His-DDX1 was evaluated using 10% SDS-PAGE and Coomassie-blue staining. Peak fractions (2 ml) containing His-DDX1 were subjected to gel filtration using a Superdex® 200 10/300 column pre-equilibrated in DDX1 storage buffer (20 mM Tris, pH 8; 200 mM KCl; 5 mM MgCl_2_; 2 mM DTT) at a 0.5 ml/min flow rate for the duration equivalent to 1.1 CV. All purification steps were performed on the ÄKTA Purifier FPLC system (Cytiva). The elution profile of His-DDX1 (750 μl fractions) was monitored by SDS-PAGE and Coomassie-blue staining. His-DDX1-containing fractions were supplemented with glycerol (final concentration 10%), aliquots (25 μl) were flash-frozen in liquid nitrogen and stored at –80°C until use.

### Expression and purification of recombinant *D. melanogaster* belle

The coding sequence of the *D. melanogaster* DDX3X homologue (belle, CG9748) was cloned by synthesis with an N-terminal MBP and a C-terminal His-tag (BioCat, Heidelberg) and expressed in *E. coli* BL21(DE3) pLysS (Invitrogen) in LB medium supplemented with ampicillin (50 μg/ml) and chloramphenicol (50 μg/ml). Protein expression was induced at OD600 = 0.6 using IPTG (0.5 mM) at 17°C for 18 h. Bacteria were harvested by centrifugation (8 min at 3500 g). Cell pellets were resuspended in 10 ml His-buffer A (20 mM Tris, pH 8; 5 mM imidazole; 500 mM KCl; 1 mM DTT) supplemented with protease inhibitors (Roche). Cell disruption was performed by sonication on ice (5 cycles, 60 s work at 60%/30 s pause). Lysates were cleared by centrifugation for 10 min using 21 000 g at 4°C. Cleared lysates were loaded onto Ni-NTA His Trap High Performance columns (5 ml, Cytiva) that were pre-equilibrated in His-buffer A. Columns were washed with buffer A followed by elution of recombinant protein in His-buffer B (20 mM Tris, pH 8; 500 mM imidazole; 500 mM KCl; 1 mM DTT). The elution profile of MBP-belle-His was evaluated using 10% SDS-PAGE and Coomassie-blue staining. Peak fractions (2 ml) containing MBP-belle-His were subjected to gel filtration using a Superdex® 200 10/300 column pre-equilibrated in belle storage buffer (50 mM Tris, pH 8; 300 mM KCl; 1 mM DTT) at a 0.5 ml/min flow rate for the duration equivalent to 1.1 CV. All purification steps were performed on the ÄKTA Purifier FPLC system (Cytiva). The elution profile of MBP-belle-His (750 μl fractions) was monitored by SDS-PAGE and Coomassie-blue staining. Belle-containing fractions were supplemented with glycerol (final concentration of 10%), aliquots were flash-frozen in liquid nitrogen and stored at –80°C until use.

### Use of recombinant DHX36

Lyophilized recombinant human DHX36 protein fragment (GST-DHX36_136-988_-His) was commercially obtained (ProteoGenix, PX-P6086). The lyophilised protein was resuspended in 1× PBS supplemented with 1 mM DTT and 10% glycerol, flash-frozen in liquid nitrogen and kept at –80°C until use.

### Expression and purification of recombinant AlkB versions

Expression vectors encoding the cDNA of truncated AlkB and AlkB D135S (pET30a-AlkB and pET30a-AlkB-D135S (40)) were a gift from Tao Pan (Addgene plasmids #79051, #79050). Each expression vector was transformed in competent *E. coli* BL21(DE3) pLysS and grown at 37°C until an OD600 of 0.6–0.8 was reached. Expression was induced with 1 mM IPTG and 5 μM FeSO_4_ for 4 h at 30°C. Bacteria were pelleted, washed with 1× PBS and resuspended in lysis buffer (10 mM Tris, pH 7.4; 300 mM NaCl; 1 mM DTT; 5% glycerol; 10 mM imidazole; 0.2% protease inhibitors). After sonication, the cleared lysate was run on a HisTrap FF column (1 ml, Cytiva) equilibrated with buffer A (10 mM Tris, pH 7.4; 300 mM NaCl; 1 mM DTT; 5% Glycerol; 10 mM imidazole). To remove nucleic acids bound to AlkB, the column was washed with a high salt buffer (10 mM Tris pH 7.4, 1 M NaCl, 1 mM DTT, 5% glycerol). The protein was eluted with buffer B (10 mM Tris, pH 7.4; 300 mM NaCl; 1 mM DTT; 5% glycerol; 500 mM imidazole) and fractions containing AlkB were concentrated with Vivaspin 10 000 MWCO (Sartorius). The concentration of purified proteins was calculated by comparison with BSA standards on a Coomassie brilliant blue stained SDS-PAGE; image analysis was done with ImageQuant TL (Cytiva). Purified enzymes were diluted with buffer B to 500 μM, snap-frozen and stored in aliquots at –80°C. Repeated freezing-thawing cycles did not affect the activity of the protein.

### AlkB demethylation activity assay

Purified AlkB_wt_ and AlkB_D135S_ were tested for demethylation activity on RNA oligonucleotides harbouring a 5′-end modification. In a reaction volume of 25 μl, 50 or 250 pmol AlkB were incubated with 2 pmol of a synthetic RNA oligonucleotide carrying m^1^A at its 5′ end (IBA Sciences) previously ^32^P-labelled with T4 PNK (NEB) in demethylation buffer (50 mM HEPES–KOH, pH 8; 1 mM α-ketoglutaric acid; 2 mM sodium ascorbate; 75 μM (NH_4_)_2_Fe(SO_4_)_2_; 50 μg/ml BSA) (30), 250 ng total RNA extracted from HAP1 cells, and 40 U murine RNase inhibitor (NEB) for 3 or 30 min at 25°C. Similarly, 50 or 250 pmol of AlkB_D135S_ were used to treat 2 pmol ^32^P-labelled m^1^G-modified RNA oligonucleotide (kindly provided by R. Micura/University of Innsbruck) for 3 or 30 min. Aliquots were withdrawn at indicated time points, guanidine hydrochloride was added (final 166 mM), and samples were further processed for TLC as previously described (41). RNA integrity was verified by denaturing PAGE.

### AlkB demethylation of purified tRNA

One microgram of PAGE-purified tRNA from HeLa cells was incubated with a combination of recombinant AlkB_wt_ and AlkB_D135S_ (250 pmol each) in demethylation buffer in a total volume of 25 μl for a duration of 45 min. As control, purified tRNA was incubated in demethylation buffer without AlkB. 400 μl of stop buffer (300 mM sodium acetate, pH 5.0; 0.1% SDS; 1 mM EDTA) was added to demethylation reactions, followed by RNA extraction using acidic P/C/I and precipitation using isopropanol. tRNAs were resuspended in water and used for the preparation of 3′-tracer tRNAs.

### ATP hydrolysis assays

Assays were performed as previously described for DDX1 (42), DDX3X (37), DDX5 (39), eIF4A1 (using condition B from (43)) and DHX36 (44). Briefly, recombinant proteins (500 nM) were mixed with RNA (1 μM, see oligonucleotides) and 2 mM equimolar ATP-MgCl_2_ stock solution followed by incubation at 37°C for 30 min. Reactions were quenched by addition of EDTA to 100 mM and placing samples on ice, followed by colorimetric quantification (620 nm) of released phosphates using Green Reagent (Enzo Life Sciences).

### Electro-mobility shift assays (EMSA)

Full-length tRNAs as well as tRNAs (full-length or affinity-captured tRNAs) that were subjected to rANG hydrolysis were tested for binding to MBP-DDX3X or eIF4A1-GST using EMSA. ^32^P-labelled tRNAs or synthetic tRNA-Lys^UUU^ as well as 3‘-tracer tRNAs were resuspended in DDX3X helicase buffer (40 mM Tris-Cl, pH 8; 50 mM NaCl; 0.5 mM MgCl_2_; 2 mM DTT; 0.01% NP40) (37) or eIF4A1 binding buffer (25 mM Tris, pH 7.5; 1 mM DTT; 100 mM KCl; 5 mM MgCl_2_) (45). MBP-DDX3X and GST-eIF4A1 aliquots were thawed on ice and dialysed into either helicase (DDX3X) or binding buffer (eIF4A1) using 0.025 μm MCE nitrocellulose membranes (Millipore). Increasing amounts of recombinant proteins were pre-incubated with equimolar concentrations of AMP-PNP/MgCl_2_ (2 mM) for 5 min at 37°C. Binding reactions were initiated by addition of either ^32^P-labelled tRNAs or 3′-tracer tRNAs, followed by incubation for 30 min at 37°C. Formed RNPs were exposed to UV light (254 nm) for 3 min (Stratagene). Equal volumes of 2x native loading dye (25% sucrose, 5 mM MgCl_2_) were added to binding reactions and RNPs were separated using nPAGE (6%) in 0.5× TB supplemented with 5 mM MgCl_2_. Gels were exposed to storage phosphor screens (Cytiva) for ≤ 2 h and imaged using a Typhoon imager (Cytiva). For the quantification of DDX3X affinities to tRNA-Lys^UUU/CUU^, the percentages of detectable DDX3X-shifted tRNA-Lys^UUU/CUU^ signal from triplicate EMSAs were determined using Biorad Image Lab, and values were plotted against the molar concentration of MBP-DDX3X. The dissociation constant (*K*_d_) was calculated by applying the equation for one site-specific binding curve using Prism 5.

### RNA helicase activity assays (^32^P-labelled 3′-tracer tRNAs)

For testing cytoplasmic protein extracts, 4 pmol of 3′-tracer tRNAs were resuspended in common RNA helicase buffer (10 mM Tris, pH 8.0; 50 mM KCl; 5 mM MgCl_2_; 10 mM DTT). Aliquots of cytoplasmic protein extracts were thawed on ice and dialysed against common RNA helicase buffer using 0.025 μm MCE nitrocellulose membranes (Millipore). Protein concentrations were measured using Bradford and 0.5 to 10 μg of dialysed cytoplasmic protein extract were used per RNA helicase reaction. For testing of recombinant proteins, 4–6.5 pmol of 3′-tracer tRNAs or 3′-tracer tRNAs-Gly^GCC/CCC^ were resuspended in either DDX3X helicase buffer (37), eIF4A1 helicase buffer (45) or DDX5 helicase buffer (39) and placed on ice. MBP-DDX3X, MBP-DDX3X_DQAD_, eIF4A1-GST, MBP-DDX5-GST, His-DDX1 or GST-DHX36_136-988_ aliquots were thawed on ice and dialysed against the respective helicase buffers (canonical or non-canonical) using 0.025 μm MCE nitrocellulose membranes (Millipore). Before starting the RNA helicase reaction, protein extracts or recombinant proteins were pre-incubated either with equimolar amounts of ATP/MgCl_2_ (2 mM) or AMP-PNP/MgCl_2_ for 5 min at 37°C. RNA helicase activity was initiated by addition of 5′ end-labelled tRNA duplexes, 3′-tracer tRNAs or 3′-tracer tRNAs-Gly^GCC/CCC^, followed by incubation at 37°C for the indicated times. Reactions were stopped by placing reactions on ice and the addition of 2x native loading dye (25% sucrose; 5 mM MgCl_2_). Reactions were separated by nPAGE (10%) in 0.5× TB supplemented with 5 mM MgCl_2_. Gels were exposed to storage phosphor screens for ≤ 2 h and imaged using a Typhoon imager (Cytiva). For time-course experiments, aliquots were removed at indicated times, quenched with 2x native loading dye on ice and separated by nPAGE (6%) using 0.5× TB supplemented with 5 mM MgCl_2_. The percentage of displaced 3′ tsRNAs was calculated as follows: Frac^SS^ = Intensity^SS^/(Intensity^DS^ + Intensity^SS^), ss = single-stranded, ds = double-stranded. To calculate unwinding rates for DDX3X reactions, time-course experiments were performed in three replicates and as technical duplicates. After averaging the fraction of duplexes (from technical duplicates) that were not unwound by DDX3X activity, data was fitted to the integrated rate law for a homogeneous first-order reaction (Frac^SS^ = *A*(1 – e^−kt^)) as described before (46), by applying the equation for one-phase exponential association using Prism 5. Statistical analysis was performed by applying an unpaired, two-tailed, t-test on replicate data representing separated 3' tsRNAs (in percent). For experiments using AlkB-treated tRNAs, signals originating from 3' tsRNA displacement at time point zero were subtracted from subsequent time points prior to statistical analysis.

### RNA helicase activity assays (‘cold’ 3′-tracer tRNAs)

For testing tsRNA integrity derived from 3′-tsRNAs, total tRNA was incubated with rANG, PNK treatment was omitted, followed by precipitation using isopropanol in the presence of 5 mM MgCl_2_. About 4 pmol of ‘cold’ 3′-tracer tRNAs were resuspended in common RNA helicase buffer (10 mM Tris, pH 8.0; 50 mM KCl; 5 mM MgCl_2_; 10 mM DTT). Aliquots of cytoplasmic protein extracts from different cell lines were thawed on ice and dialysed against common RNA helicase buffer using 0.025 μm MCE nitrocellulose membranes (Millipore). Protein concentrations were measured using Bradford, and 5 μg of dialysed cytoplasmic protein extracts were used along with 400 fmol ‘cold’ 3′-tracer tRNAs per activity assay reaction for 30 min at 37°C. Reactions were stopped by addition of Proteinase K to 200 μg/ml, followed by incubation for another 10 min at 37°C. RNA was extracted from reactions using acidic phenol (Roth) followed by ethanol precipitation in 300 mM NaOAc, pH 5. Sample RNAs were processed for denaturing PAGE and NB as described above.

### RNA helicase-mediated annealing assays

3′-tracer tRNAs were heat-denatured in DDX3X helicase buffer (37) at 75°C for 2 min to generate single-stranded RNAs, and placed on ice. MBP-DDX3X was thawed on ice and dialysed against DDX3X helicase buffer. Annealing reactions were initiated by addition of denatured 3′-tracer tRNAs, followed by incubation at 37°C. Aliquots were removed at indicated times and quenched with 2× native loading dye (25% sucrose, 5 mM MgCl_2_) on ice. Reactions were separated by nPAGE (10%) in 0.5× TB supplemented with 5 mM MgCl_2_. Gels were exposed to storage phosphor screens for ≤ 2 h and imaged using a Typhoon imager (Cytiva).

### iCLIP analysis

Raw fastq sequences resulting from DDX3X iCLIP and matched RNA-Seq data were downloaded from the Gene Expression Omnibus (GEO) GSE70804 and subjected to a mapping pipeline (written in Snakemake) tailored to the accurate quantification of ncRNA (47). Specifically, reads were trimmed for adapters and sequence quality (PHRED > 30) using Trim Galore! (http://www.bioinformatics.babraham.ac.uk/projects/trim_galore/). Reads were then mapped sequentially to ncRNAs using Bowtie allowing a maximum of two mismatches (48). Mapping was first performed to representative snRNA and rRNA sequences downloaded from NCBI (49), followed by mapping to mature tRNA sequences (3′ CCA and 5′ G added), then to immature tRNA sequences (containing introns and flanking 50 nt), and last to the human mitochondrial chromosome. Remaining reads were mapped to the human genome. tRNA annotations were downloaded from UCSC table browser, which sources the annotations from GtRNAdb (50). A custom-made script was used to resolve those reads that multi-mapped to individual tRNA gene sequences, since tRNA genes within the same isoacceptor do have high sequence similarities. Crucially, since this level of sequence similarity is not identical between tRNA isoacceptors, quantification may be biased towards particular tRNA isoacceptors with less sequence similarity between their genes. This mapping strategy is data-driven and aimed at creating groups of genes that commonly multi-map. Even though aimed at resolving tRNA isoacceptors, the strategy occasionally results in merging two or more tRNA isoacceptors. To do so, first, multi-mapping reads were labelled with the tRNA isoacceptor type to which they mapped, followed by only keeping reads with the least mismatches. Consensus isoacceptor groups were then derived based on the percentage of multi-mapping reads the user wishes to retain from each sample. For this mapping, 90% was chosen. For deriving positional information from iCLIP data, crosslink positions along a mature tRNA sequence were assigned based on the mode of read starts –1. In case of no mode, read starts were randomly chosen from possible positions indicated by multi-mapping reads. To generate tRNA metaprofiles, reads at crosslink positions from each group were summed and divided by all reads mapping to tRNAs in a given sample. All plotting was produced in R, using data.table (https://dplyr.tidyverse.org/reference/dplyr-package.html), dplyr (https://dplyr.tidyverse.org/) and ggplot2 (51).

### Small RNA sequencing

^32^P-labelled tRNA duplexes or RNAs migrating at the mass of ^32^P-labelled 3′ tsRNAs were excised from native PA gels, and RNA was extracted from crushed gel pieces using RNA extraction buffer (300 mM sodium acetate, pH 5.0; 0.1% SDS; 1 mM EDTA) after overnight incubation at 4°C. After precipitation of ^32^P-labelled RNAs using isopropanol, cDNA libraries were prepared for small RNA sequencing using NEBNext multiplex small RNA Library Prep set 2 for Illumina (NEB), following the manufacturer's instructions. Following cDNA synthesis and omitting the 75°C denaturation step, ^32^P-labelled RNA-cDNA hybrids were treated with RNase H (25 U, NEB) for 30 min at 37°C, followed by PCR amplification. PCR amplicons were separated from remaining ^32^P-labelled RNA fragments by nPAGE in 0.5× TB supplemented with 5 mM MgCl_2_, normalised for quantity and submitted for sequencing on an Illumina MiSeq (PE75 mode) or NovaSeq S2 (PE150 mode) or NextSeq550 (SR150 mode).

### Analysis of small RNA sequencing data

Reads were trimmed for adapter sequences and low-quality bases with *q*-score < 30 were removed from the 3' end using the cutadapt software. Mapping of trimmed reads to a collapsed tRNA annotation was performed as previously published (52). In short, collapsed human tRNA genomic space with added CCA sequences on the 3' end was used to create the respective Bowtie2 index. After mapping with Bowtie2, the abundance of tRNA-derived reads was extracted using Samtools idxstats, normalised to library sequencing depth, and the normalised tRNA abundance per library was plotted as a heat map using python Seaborn library. In order to calculate fold-changes, the normalised tRNA abundance originating from DDX3X-treated sRNA libraries were divided by the normalised tRNA abundance originating from heat-denatured sRNA libraries from the respective replicate experiment. The obtained values were plotted as a heat map using python Seaborn library.

### Command line parameters

cutadapt: cutadapt -q 30 -a AGATCGGAAGAGCACACGTCTGAACTCCAGTCAC input.fastq > output_trimmed.fastq

Bowtie2: bowtie2 –no-unal –no-1mm-upfront -D 15 -R 2 -N 0 -L 10 -i S,1,1.15 -x index -U sample.fastq -S output.sam 2 > sample_mapping_report.txt

Samtools idxstats: samtools idxstats sample_sorted.bam > sample_idxstats.txt

### Oligonucleotide sequences (5′-3′)

ATPase assays

BG0147: AGAUCGGAAGAGCACACGUCUAGUUCUACAGUCCGACGAUCNNGGUUNN

Northern blotting

5′ tRNA-GlyGCC/CCC (AD0006): TCTACCACTGAACCACCAAT

3′ tRNA-GlyGCC (AD0007): TGGTGCATTGGCCGGG

5′ tRNA-GluCUC/UUC (AD0008): GAATCCTAACCACTAGACCAC

5′ tRNA-ValAAC (AD0138): GAACGTGATAACCACTACACTACGGAAA

Affinity capture of specific tRNAs

tRNA-Gly^GCC/CCC^ (AD0023): 5'-Amino-Modifier C6-AGGCGAGAATTCTACCACTGAACCACC

tRNA-Lys^UUU/CUU^ (AD0213): 5'-Amino-Modifier C6-TCTGATGCTCTACCGACTGAGCTATCC

Cloning of shRNAs into pLKO.1

shRNA_DDX3X_fwd (AD0156): CCGGCGGAGTGATTACGATGGCATTCTCGAGAATGCCATCGTAATCACTCCGTTTTTG

shRNA_DDX3X_fwd (AD0157): AATTCAAAAACGGAGTGATTACGATGGCATTCTCGAGAATGCCATCGTAATCACTCCG

AlkB substrate RNAs

m^1^AUGCACUUGGACGAACCAGAGUGUAGCUUAA

m^1^GGCGCAGCGGAAGCGUGCUGGGCCCA

Non-targeting siRNA pool

UAGCGACUAAACACAUCAA

UAAGGCUAUGAAGAGAUAC

AUGUAUUGGCCUGUAUUAG

AUGAACGUGAAUUGCUCAA

hDDX3X-targeting siRNA pool

GCAAAUACUUGGUGUUAGA

ACAUUGAGCUUACUCGUUA

CUAUAUUCCUCCUCAUUUA

GGUAUUAGCACCAACGAGA

hDDX5-targeting siRNA pool

CACAAGAGGUGGAAACAUA

GUGAUGAGCUUACCAGAAA

GCAAAUGUCAUGGAUGUUA

GGAUGUGUGUCAUGACGUA

## RESULTS

### Production of ‘tracer tRNAs’ for the *in vitro* dissection of tsRNA biogenesis

**Figure 1.**
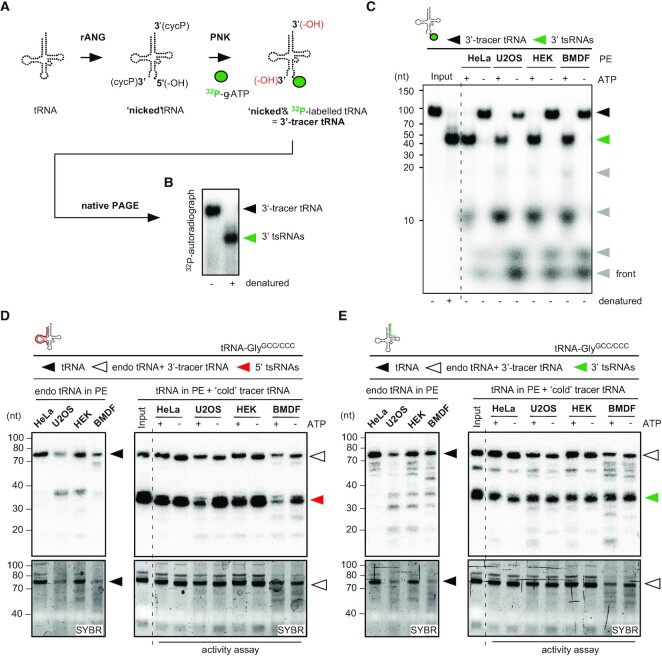
Cytoplasmic protein extracts contain activities which separate and degrade 3‘-tracer tRNAs. (**A**) Schematic representation of enzymatic reactions for the production of 3′-tracer tRNAs. ^32^P-γ-ATP-labelling of tRNAs that were hydrolysed by recombinant ANG. Position of the label (^32^P) on 3' tsRNAs is marked as a green dot. rANG, recombinant Angiogenin; PNK, T4 polynucleotide kinase; cycP, 2′-3′ cyclic phosphate; -OH in black, hydroxyl moiety as result of rANG activity; -OH in red, hydroxyl moieties as result of PNK activity. (**B**) Representative nPAGE of ^32^P-γ-ATP-labelled 3′-tracer tRNAs before and after heat denaturation. Black arrowhead, duplex of 3′-tracer tRNAs; green arrowhead, 3′ tsRNAs. (**C**) 3′-tracer tRNAs (8 nM final) were incubated with cytoplasmic protein extracts (2 μg) obtained from different cell lines in the presence or absence of 2 mM ATP. Reactions were separated by nPAGE and 3′ tsRNA signals were collected by exposing PAA gels to phosphor-imaging plates for ≤ 2 h. Black arrowhead, 3′-tracer tRNAs; green arrowhead, 3′ tsRNAs; grey arrowheads, signals from 3′ tsRNA degradation; PE, protein extract. (**D**) ‘Cold’ 3′-tracer tRNAs (8 nM final) were incubated with cytoplasmic protein extracts (2 μg) obtained from different cell lines in the presence or absence of 2 mM ATP. RNA was extracted from activity assays and separated by on denaturing PAGE followed by NB for tRNA-Gly^GCC/CCC^ using a probe against the 5′ moiety (right panel). To reveal the tRNA and 5′ tsRNA content of cytoplasmic protein extracts, total RNAs extracted from 2 μg protein extracts were probed in parallel (left panel). Top panels, NB; bottom panels, SYBR staining; black arrowhead, tRNA-Gly^GCC/CCC^ in protein extracts; white arrowhead, combined signal from tRNAs (protein extracts) and ‘cold’ 3′-tracer tRNAs; red arrowhead, 5′ tsRNAs. (**E**) ‘Cold’ 3′-tracer tRNA assay as described in (D) but probed for the 3′ moieties of tRNA-Gly^GCC/CCC^. Top panels, NB; bottom panels, SYBR staining; black arrowhead, tRNA-Gly^GCC/CCC^ in protein extracts; white arrowhead, combined signal from tRNAs (protein extracts) and ‘cold’ 3′-tracer tRNAs; green arrowhead, 3′ tsRNAs.

Previous biochemical attempts aimed at identifying tsRNA-interacting proteins mostly utilised synthetic 5′ and/or 3′ tsRNAs as bait molecules (8,10,11,21,53) while approaches using native tsRNAs remain the exception (54). Furthermore, native tRNAs substrates and assay conditions that would allow for the molecular dissection of the biogenesis of stress-induced tsRNAs have not been reported yet. We reasoned that such an experimental system should be biochemically tractable *ex vivo*, open to genetic manipulation of the individual reactants and relatively easy to use when monitoring the processing of tRNAs into tsRNAs *in vitro*. Most importantly, as post-transcriptional modifications were shown to modulate tRNA cleavage events (55,56), the experimental system should incorporate endogenously modified tRNAs as substrates for the activity of anticodon nucleases and for activities binding to and acting on ‘nicked’ tRNAs. To produce endogenously modified and, importantly, structured tRNAs with a break in the AC-loop as a mimic for stress-induced tRNA fragmentation, bulk tRNAs were purified from human cells, followed by 5′ or 3′ end-labelling using γ-^32^P-ATP or 3′,5′ [5′-^32^P]pCp, respectively. ^32^P-labelled tRNAs were heat-denatured, re-folded in the presence of Mg^2+^-ions and incubated with recombinant ANG (rANG). Native polyacrylamide gel electrophoresis (nPAGE) was used to monitor the integrity of rANG-processed tRNAs (Supplementary Figure S1A). The results showed that ^32^P-labelled tRNAs could be maintained as duplexes after rANG-mediated processing since heat denaturation prior to nPAGE resulted in faster migration of 5′ tsRNA moieties representing only a fraction of ³²P-labelled RNAs (Supplementary Figure S1B). In contrast, rANG incubation of 3′ end-radiolabelled tRNAs resulted in the loss of ³²P-label, indicating that rANG removed the non-templated CCA at 3′ ends of tRNAs (Supplementary Figure S1C), which confirmed previous observations (57), but excluded 3′ end-labelling as an option for following the fate of ‘nicked’ tRNAs. ANG-mediated RNA hydrolysis creates 3′ RNA moieties containing a 5′ hydroxyl while 5′ RNA moieties contain a 2′, 3′-cyclic phosphate (cycP) at their 3′ ends (9,58). Notably, ANG displays some tRNA substrate specificity (23,57). To label only those tRNAs that were targeted by ANG, purified human tRNAs were exposed to rANG followed by ³²P-labelling at 5′ hydroxyl groups that were created at 3′ tsRNA moieties after ANG-mediated hydrolysis (Figure 1A). nPAGE revealed that those tRNAs containing rANG-mediated ‘nicks’ migrated as duplexes, which consisted of ³²P-labelled 3′ tsRNAs bound to non-labelled 5′ tsRNAs (Figure 1B). This indicated that specifically labelling tsRNA moieties after rANG-processing allowed tracing the fate of ANG-processed substrates (termed 3‘-tracer tRNAs from here on), while leaving tRNAs that were non-ANG substrates invisible to detection by phospho-imaging. To determine the identity of 3‘-tracer tRNAs, they were eluted from nPAGE, linker-ligated,reverse-transcribed, and cDNAs corresponding to the lengths of tsRNAs (20–40 nucleotides) were sequenced (sRNAseq). Read mapping was performed to non-redundant tRNA sequence clusters (52) to avoid multi-mapping biases of short or post-transcriptionally modified tRNA-derived reads. Quantification of the relative abundance of tRNA-derived reads revealed that only a few tRNA isoacceptors, all of which were previously reported to be ANG-substrates (23,57), contributed substantially to 3‘-tracer tRNAs (Supplementary Figure S1D). These combined results showed that duplex structures of ANG-processed tRNAs can be maintained as 3‘-tracer tRNAs *in vitro*, opening up the possibility for dissecting their molecular processing into individual tsRNA moieties through experimental manipulation.

### Mammalian cell extracts contain activities which separate and degrade 3‘-tracer tRNAs

Specific tsRNAs can be detected in the cytoplasm of acutely stressed cells (54,59), indicating that stress-induced tsRNA biogenesis is likely a cytoplasmic process. To determine if 3‘-tracer tRNAs can be used to reveal cellular activities that affect the structural integrity of rANG-processed tRNAs, 3′-tracer tRNAs were incubated with cytoplasmic protein extracts from mammalian cell lines and analysed by nPAGE. This revealed a faster migration of labelled 3′ tsRNAs that was ATP-dependent, albeit with noticeable loss of label from 3′ tsRNAs, which was also cell line-dependent (Figure 1C). Notably, PNK treatment of RNAs containing 3′ cycP will result in dephosphorylation of these moieties, hence 3′-tracer tRNAs produced in this way are likely to become accessible to 3′-5′ exonuclease activities. To address if the change in migration of labelled 3′ tsRNAs from 3′-tracer tRNAs could have been the result of degrading non-labelled 5′ tsRNAs, mature human tRNAs were ³²P-labelled at 5′ moieties before exposure to rANG, incubation with cytoplasmic protein extracts and analysis by nPAGE. The results showed an ATP-dependent mobility increase of labelled 5′ moieties originating from 5′-tracer tRNAs, the migration pattern of which was comparable to those of heat-denatured 5′-tracer tRNAs (Supplementary Figure S1E). Furthermore, northern blotting (NB) of ‘cold’ (non-labelled) 3′-tracer tRNAs for both 5′ and 3′ tsRNA-Gly^GCC/CCC^ after exposure to cytoplasmic protein extracts revealed either equal levels of both 5′ and 3′ tsRNAs (HeLa-derived extracts), or lower levels of 5′ tsRNAs (extracts derived from other cell lines (Figure 1D, E)). In addition, analysis of the integrity of the 3′ moieties in 3′-tracer tRNAs using denaturing PAGE, indicated a gradual loss of ³²P-label and also 3′-terminal nucleotides after exposure to cell extract, both of which was protein concentration-dependent (Supplementary Figure S1F) and indicated cellular activities which degraded 3′ tsRNAs from their 3′ ends. Of note, the levels of label loss from 3′-tracer tRNAs versus maintenance of 3′-tracer tRNA integrity were dependent on the buffer composition into which cytoplasmic protein extracts were dialysed before the activity assays on 3′-tracer tRNAs. This observation suggested the presence of competing cellular activities acting on 3′-tracer tRNAs (degrading versus unwinding 5′ from 3′ tsRNAs), the balance of which was determined by the particular buffer composition (Supplementary Figure S1G). These results indicated that cytoplasmic protein extracts contained ATP-dependent activities with the ability of separating 5′ tsRNAs from 3′ tsRNAs while partially degrading both tsRNA moieties from their respective 3′ ends.

### Stress-induced tsRNAs are associated with RNPs containing RNA helicases

**Figure 2.**
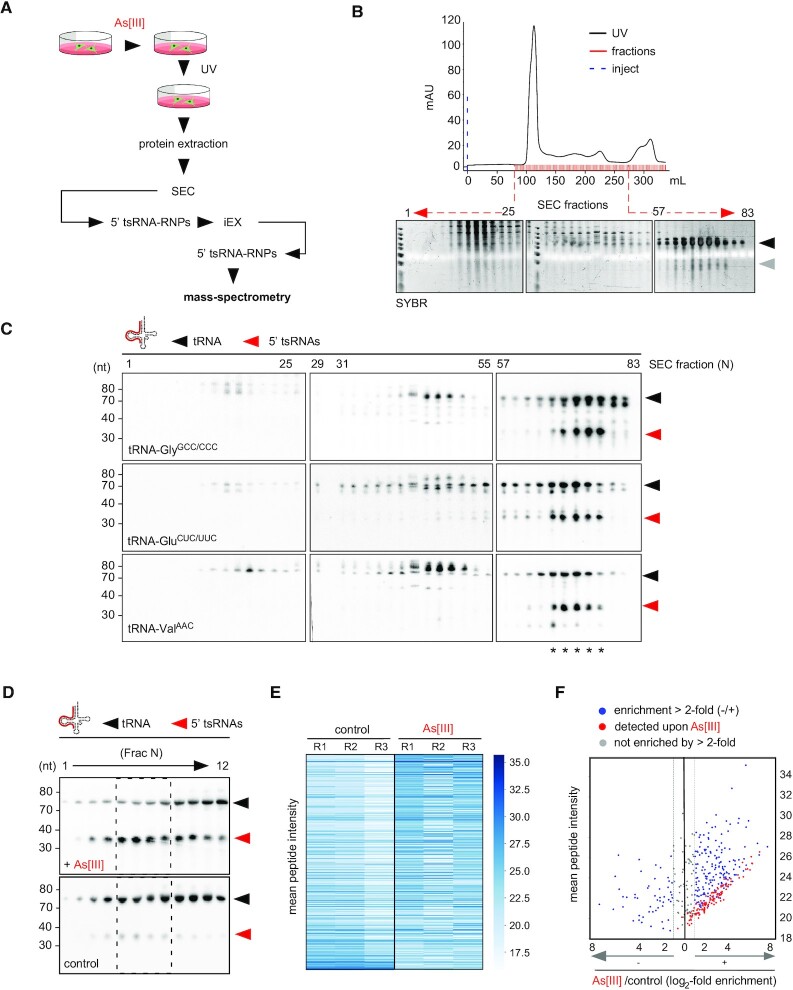
Biochemical fractionation identifies proteins co-migrating with 5′ tsRNAs after the stress response. (**A**) Schematic representation of the workflow for the biochemical fractionation resulting in the enrichment and identification of tsRNA-containing RNPs. As[III], inorganic sodium meta-arsenite; UV, ultraviolet light treatment; SEC, size exclusion chromatography; IEX, ion exchange chromatography; RNPs, ribonucleoprotein particles. (**B**) Representative size exclusion chromatogram for RNPs originating from cell extracts obtained from HEK293 cells after As[III] exposure (black line, UV trace; blue dashed line, injection of sample). RNA from every second fraction of denoted SEC fractions (red marks) was extracted, separated by urea-PAGE and stained with SYBR-Gold; mAU, milli absorbance units; black arrowhead, mature tRNAs; grey arrowhead, small RNAs including tsRNAs. (**C**) NB of total RNA extracted from representative SEC fractions as shown in (B) using a probe against the 5′ end of tRNA-Gly^GCC/CCC^, tRNA-Glu^CUC/UUC^ and tRNA-Val^AAC^. Black arrowheads, mature tRNAs; red arrowheads, 5′ tsRNAs; asterisks, SEC fractions that were pooled and further subjected to ion-exchange chromatography. (**D**) Representative NB of total RNA extracted from fractions (*N* = 12) that were obtained from subjecting 5′ tsRNA-containing SEC fractions to ion-exchange chromatography (IEX) using a probe against tRNA-Gly^GCC/CCC^. Upper panel represents a blot obtained after IEX on SEC fractions from cells exposed to sodium meta-arsenite (+ As[III]). Lower panel represents blot obtained after IEX on SEC fractions from control cells. Dashed boxes mark fractions (5–8) that were analysed for protein content by mass spectrometry. Black arrowheads, mature tRNAs; red arrowheads, 5′ tsRNAs. (**E**) Heat map representing mean peptide intensity values of proteins detected by mass spectrometry across replicate experiments (R1–3) on control and As[III]-exposed protein extracts. Peptide intensity values (y-axis) were sorted in a descending manner according to their log_2_- transformed enrichment upon As[III] exposure. (**F**) Scatter plot of proteins identified in IEX fractions. Mean peptide intensities of identified proteins (y-axis) from replicate experiments were plotted against their log_2_-transformed enrichment after As[III] exposure. Blue dots depict peptides enriched more than 2-fold (log_2_> 1, represented by vertical grey lines) in controls (log_2_ < –1) and after As[III] exposure (log_2_ > 1). Red dots represent peptides specifically detected upon As[III] exposure. Grey dots represent peptides enriched less than 2-fold in both experimental conditions (–1 < log_2_< 1).

Previous work aimed at identifying tsRNA-interacting proteins utilised exclusively *ex-vivo* RNA affinity approaches with specific tsRNAs as bait (8,10,11,21,53,54). Notably, the value of such approaches remains limited since protein binding to a respective tsRNA species was usually forced by using exorbitant amounts of RNA bait in conjunction with ill-defined compositions of protein extracts. We reasoned that factors which bind and potentially process ‘nicked’ tRNAs should be in close physical proximity with stress-induced tsRNAs. To identify such factors *in vivo*, tsRNA-containing RNPs were enriched by biochemical fractionation and identified by mass spectrometry. To do so, HEK293 cells were transiently exposed to inorganic sodium meta-arsenite (As[III]), followed by UV-crosslinking and protein extraction under non-denaturing conditions (Figure 2A). Western blotting for phosphorylated eIF2α, a marker for the integrated stress response, verified the activation of stress response pathways upon As[III] exposure (Supplementary Figure S2A). NB for tRNA-Gly^GCC/CCC^, tRNA isoacceptors whose associated 5′ tsRNAs have often been reported as overrepresented in various data sets (18,19,23), confirmed the partitioning of tsRNAs derived from tRNA-Gly^GCC/CCC^ to the soluble protein fraction with an apparent 5′/3′ asymmetry in tsRNA levels towards the maintenance of 5′ tsRNAs (Supplementary Figure S2B). Protein extracts were fractionated using size exclusion chromatography (SEC, Figure 2B) and RNPs co-migrating with 5′ tsRNAs were traced by NB for various tRNA isoacceptors (Figure 2C). SEC fractions containing 5′ tsRNAs were pooled and further separated by ion exchange chromatography (IEX, Supplementary Figure S2C). This revealed that all 5′ tsRNAs-containing IEX fractions also contained tRNAs, albeit at different ratios. IEX fractions containing a positive 5′ tsRNA/tRNA-Gly^GCC/CCC^ signal ratio on NB (Figure 2D and Supplementary Figure S2D) were subjected to mass spectrometry. To identify proteins co-migrating with full-length tRNAs, SEC and IEX fractionations were also performed on protein extracts from cells not exposed to As[III] (controls, Figure 2D and Supplementary Figure S2D). Triplicate mass spectrometry analysis of control RNPs as well as RNPs containing more 5′ tsRNAs than parental tRNAs resulted in the identification of a total of 395 proteins in at least two out of three replicate experiments (Figure 2E and Supplementary Table S1). Among those, 267 proteins were enriched by a factor ≥ 2 over controls (Figure 2F), suggesting these proteins were either migrating with 5′ tsRNAs-containing RNPs, or increased their migration with full-length tRNAs upon As[III] exposure. GO annotation for molecular function revealed a statistically significant over-representation of ‘RNA binding’ in fractionated RNPs, supporting the notion that a combination of SEC and IEX allowed enriching for proteins with the potential to interact with tsRNAs and/or tRNAs (Supplementary Figure S2E and Supplementary Table S2). Indeed, several proteins that had previously been connected to tRNA biology (TRMT10C, RTCA, EIF2S1, EEF1G, TUFM and EIF5A) co-migrated as enriched over controls upon As[III] exposure (Supplementary Figure S2F and Supplementary Table S1). Notably, 114 proteins, many of them with the potential to bind RNA (Supplementary Figure S2G and Supplementary Table S2), were exclusively co-migrating with 5′ tsRNAs-containing RNPs, as they were not detected in the corresponding fractions collected from cells grown under control conditions (Figure 2F and Supplementary Table S1). This protein subset was manually curated to extract candidate proteins with the potential to affect tsRNA biogenesis or function. Among these, translation initiation and elongation factors (eIF3A/3B/3E and eEF1A/B2/D/G), proteins involved in energy metabolism (ENOPH1, IDH1, GAPDHS, CS, G6PD), as well as several enzymes involved in RNA processing (RNAseT2, eIF4A1/A2, DDX5/17, DDX3X, DDX39A/B) were noticed (Supplementary Figure S2H, I and Supplementary Table S1). Especially the latter group of proteins suggested the physical proximity of stress-induced 5′ tsRNAs with enzymatic activities that had the potential for separating parental tRNAs containing ANG-mediated ‘nicks’.

### Various RNA helicases colocalize with stress granules during the response to as[III]

**Figure 3.**
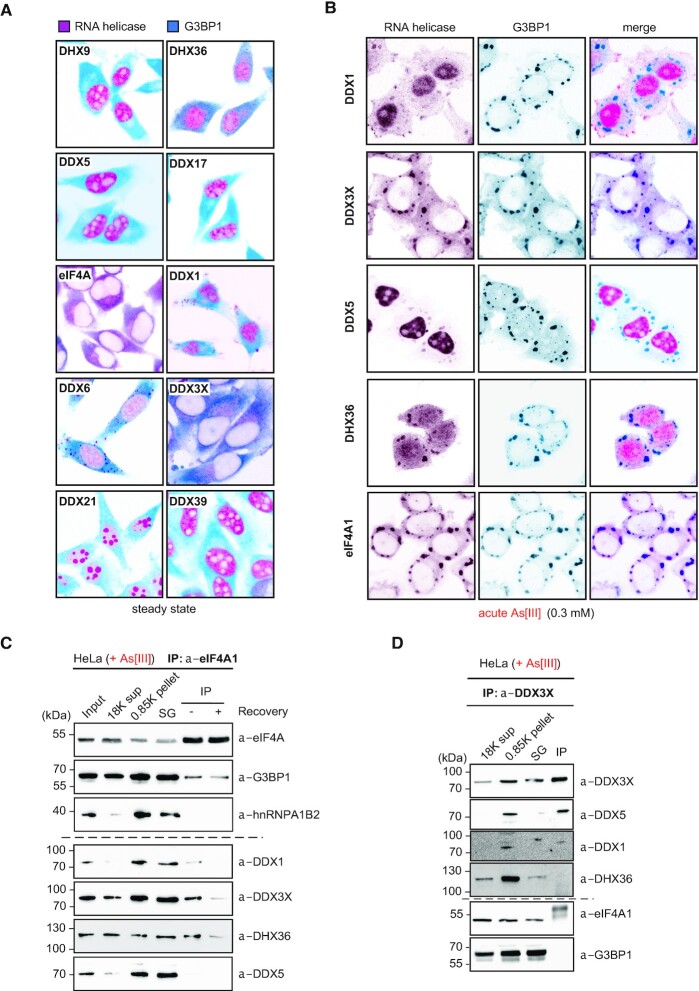
Various RNA helicases re-localize to and co-precipitate in As[III]-induced SG. (**A**) Indirect immunofluorescence images (false-coloured and merged) of HeLa cells analysed for the subcellular localization of RNA helicases (magenta), which were repeatedly identified as SG-associated and that co-migrated with 5′ tsRNAs-containing RNPs (this work) and the ubiquitous SG marker, G3BP3 (cyan), during steady-state conditions. (**B**) Indirect immunofluorescence images of HeLa cells (false-coloured) analysed for the colocalization of various RNA helicases (magenta) with G3BP1 (cyan) after acute exposure to As[III] (0.3 mM for 1 h). (**C**) Western blotting of different candidate helicases on samples obtained during the steps of SG core enrichment according to (36) and after immunoprecipitation of eIF4A1 from enriched SG cores from HeLa cells were either acutely exposed to As[III] (0.5 mM for 1 h, acute, – recovery) or were incubated in fresh medium (+ recovery) after removal of As[III]. (**D**) Western blotting of different candidate helicases on samples obtained during the steps of SG core enrichment according to (36) and after immunoprecipitation of DDX3X from enriched SG cores extracted from Hela cells acutely exposed to As[III] (0.5 mM for 1 h, acute).

Previous reports associated specific stress-induced 5′ tsRNAs with stress granule (SG) assembly (5,8,60). Furthermore, ANG colocalized with SG markers upon As[III] exposure (61) suggesting that stress-induced tRNA hydrolysis and, potentially, the processing of ‘nicked’ tRNAs occurs in proximity to SG. Using two different antibodies against endogenous ANG confirmed its colocalization with G3BP1, an ubiquitous SG marker (62), in response to As[III] exposure (Supplementary Figure S3A, B). Notably, a subset of proteins that co-migrated with 5′ tsRNAs/tRNAs-containing RNPs had been previously linked to SG (Supplementary Figure S3C and Supplementary Table S3), supporting the notion that ANG-mediated tRNA hydrolysis and processing might occur in proximity to SG. The co-migration of several DEAD box RNA helicases with 5′ tsRNAs/tRNAs-containing RNPs *in vivo* and the observation that cytoplasmic activities were able to separate ‘nicked’ tRNAs *in vitro* prompted us to investigate the subcellular localization of various RNA helicases during the stress response. Indirect immunofluorescence detection of candidate RNA helicases previously connected to SG biology, as well as of those helicases co-migrating with 5′-tsRNAs/tRNAs-containing RNPs (Supplementary Figure S3D) revealed cytoplasmic, nuclear localization and combinations thereof under steady-state conditions (Figure 3A). Notably, only a subset of these RNA helicases re-localized towards SG upon As[III] exposure (Figure 3B) suggesting functional relevance in or close to SG. Among those, eIF4A1 and DDX3X displayed absolute colocalization, while DDX1, DHX36 and DDX5 showed partial colocalization with G3BP1-positive SG. These data suggested physical proximity of ANG and specific RNA helicases at those subcellular sites that were previously connected to stress-induced tRNA hydrolysis and tsRNA biogenesis.

### Specific RNA helicases co-precipitate in SG-enriched subcellular compartments

To biochemically validate the link between SG-associated RNA helicases and As[III]-induced tsRNAs, co-fractionation experiments were repeated and western blotting was performed on IEX fractions exhibiting positive 5′ tsRNA/tRNA signal ratios on NB. The results confirmed the co-migration of eIF4A1 and DDX3X with tRNAs/tsRNAs-containing RNPs (Supplementary Figure S3E), while other RNA helicases were undetectable by western blotting. To directly determine which RNA helicases physically associated with SG, HeLa cells were exposed to As[III], followed by enrichment of SG cores (36) and immunoprecipitation of eIF4A1, an RNA helicase that is highly enriched in SG (63). Western blotting revealed the co-precipitation of eIF4A1 with the SG-marker G3BP1 and several RNA helicases (DDX1, DDX3X, DHX36, Figure 3C). Notably, reciprocal immunoprecipitation using antibodies against DDX3X revealed that SG core-enriched DDX3X can also form protein complexes with DDX5 and/or DDX1 without eIF4A1 and DHX36 (Figure 3D). These combined results indicated proximity of various RNA helicases with SGs and the products of stress-induced tRNA fragmentation raising the question whether one or more of these (candidate) RNA helicases would accept ‘nicked’ tRNAs as substrates.

### Various RNA helicases display unwinding activity towards 3‘-tracer tRNAs

**Figure 4.**
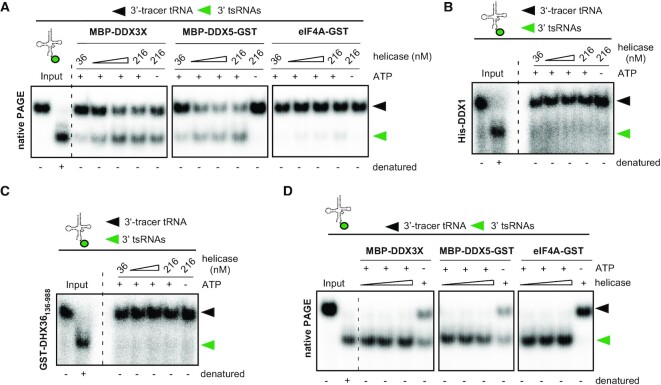
DEAD box RNA helicases accept 3′-tracer tRNAs as substrates. (**A**) Fixed-time point activity assays using increasing concentrations of recombinant MBP-DDX3X, MBP-DDX5-GST, eIF4A1-GST dialysed in their respective (canonical) RNA helicase buffer and 3′-tracer tRNAs (8 nM final) in the presence of equimolar ATP/MgCl_2_ (2 mM). As controls, the highest protein concentration was incubated with 3′-tracer tRNAs without ATP. To control for 3′-tracer tRNA and 3′ tsRNA migration, 3′-tracer tRNAs were sampled before and after heat denaturation (Input). Reactions were separated by nPAGE and ^32^P-signals were collected as described above. Black arrowhead, 3′-tracer tRNAs; green arrowhead, 3′ tsRNAs. (**B**) Fixed-time point activity assays as in (A) using increasing concentrations of recombinant His-DDX1 and 3′-tracer tRNAs in canonical RNA helicase buffer. Description of Figure details as in (A). (**C**) Fixed-time point activity assays as in (A) using increasing concentrations of recombinant GST-DHX36_136-988_ and 3′-tracer tRNAs in canonical RNA helicase buffer. Description of Figure details as in (A). (**D**) Fixed-time point activity assays using increasing concentrations of recombinant MBP-DDX3X, MBP-DDX5-GST, eIF4A1-GST and 3′-tracer tRNAs (8 nM final) in the presence of equimolar ATP/MgCl_2_ (2 mM), but in non-canonical RNA helicase buffer (buffer d, see Supplementary Figure S1G). As controls, the highest protein concentration was incubated with 3′-tracer tRNAs without ATP. Description of figure details as in (A).

DDX5, DHX36 and DDX1 had been previously connected to tRNA biology (64–66), while DDX3X and eIF4A have not been implicated in tRNA-related functions. To determine if these RNA helicases were able to catalyse the unwinding of 3′-tracer tRNAs, eIF4A1, DDX1, DDX3X and DDX5 were purified as recombinant fusion proteins (eIF4A1-GST, His-DDX1, MBP-DDX3X, MBP-DDX5-GST) while an N-terminally truncated recombinant DHX36 (GST-DHX36_136-988_) was commercially obtained. All recombinant proteins were tested for ATPase activity in published reaction buffers (canonical buffers), which had previously supported their respective activities on synthetic RNA substrates (37,39,42,44,45). All proteins hydrolysed ATP in the presence of RNA (Supplementary Figure S4A) indicating the purification of active enzymes. Notably, MBP-DDX3X and GST-DHX36_136–988_ hydrolysed some ATP in the absence of analyte RNA suggesting that these enzyme preparations contained traces of co-purified RNA or free phosphate moieties. Purified enzymes were incubated with 3′-tracer tRNAs in their respective canonical buffers and subjected to fixed time point activity assays. nPAGE revealed that MBP-DDX3X and MBP-DDX5-GST separated 3′ tsRNAs from a fraction of 3′-tracer tRNAs in a protein concentration-dependent fashion while equimolar amounts of eIF4A1-GST showed very weak activity (Figure 4A). Notably, DDX3X activity was detectable without the addition of ATP suggesting either co-purification of ATP or ATP-independent activity on 3′-tracer tRNA. In contrast, equimolar amounts of neither His-DDX1 nor GST-DHX36_136-988_ showed activity on 3′-tracer tRNAs (Figure 4B, C). Notably, testing other buffer conditions (non-canonical buffers) revealed that, in addition to MBP-DDX3X and MBP-DDX5-GST, also eIF4A-GST displayed robust RNA helicase activity on 3′-tracer tRNAs (Figure 4D). In contrast, His-DDX1 and GST-DHX36_136-988_ remained inactive in all tested buffers (Supplementary Figure S4B, C). These results indicated that, under particular *in vitro* assay conditions, more than one DEAD box RNA helicase accepted tRNAs containing ANG-mediated ‘nicks’ as substrates resulting in the separation of 5′ from 3′ tsRNAs.

### DDX3X interacts with specific tRNAs

**Figure 5.**
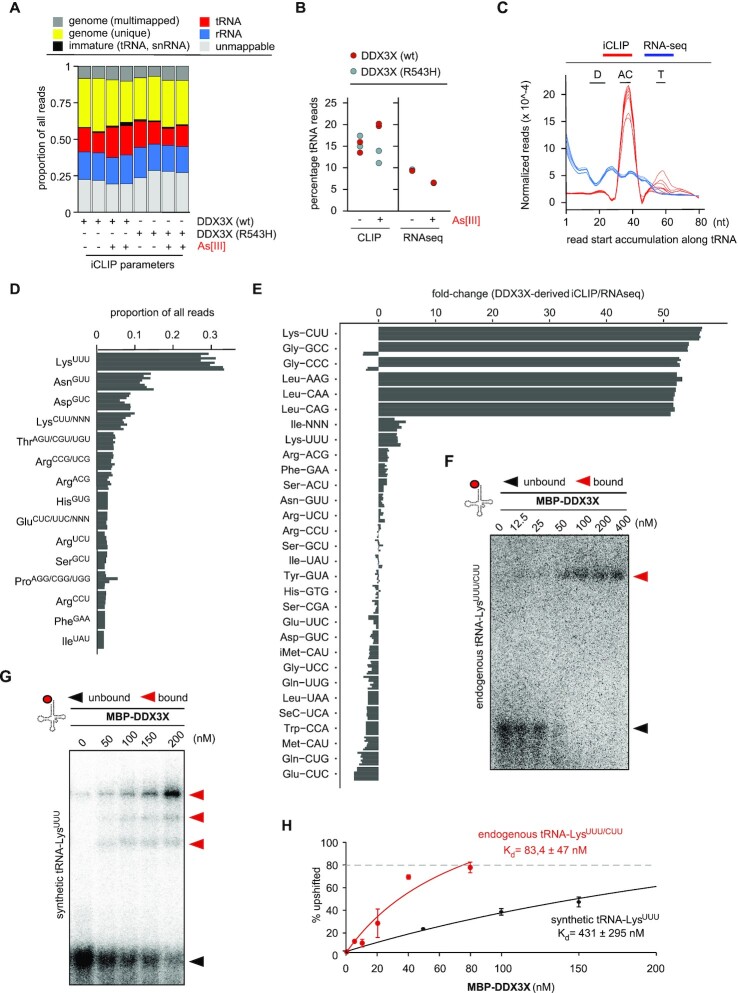
DDX3X binds to specific tRNAs. (**A**) Biotype distribution of DDX3X-derived iCLIP reads that were re-analysed from (69). Columns represent duplicate iCLIP experiments using human DDX3X (wildtype, wt) or the catalytic DDX3X mutant (R534H) under steady-state conditions and after As[III] exposure. (**B**) Changes in DDX3X-mediated iCLIP-derived tRNA reads and in tRNA expression data obtained from duplicate experiments using DDX3X or DDX3X (R534H) during steady-state conditions and after iAs exposure (69). Normalised abundance of tRNA-derived reads from iCLIP (left panel) or whole RNA-sequencing experiments (right panel) are shown. (**C**) Metaplot depicting positional information of DDX3X-mediated iCLIP signatures on tRNA sequences obtained from (69). The 5′ nucleotide positions from individual tRNA-derived reads were determined using all human tRNA genes and plotted against their abundance (red). As a comparison, tRNA-derived reads from total RNA-seq data are shown (blue). Individual lines represent replicate experiments. Letters indicate the positions of D-, anticodon- (AC) and T- loops within tRNA sequences. (**D**) Plot representing normalised abundance of DDX3X-derived iCLIP reads obtained from each experiment described in (69) and after mapping deposited data to tRNA-derived sequences. Individual rows at each tRNA isoacceptor level represent the proportion of tRNA-derived reads among all mapped tRNA sequences in replicate iCLIP experiments. (**E**) Plot representing fold-changes (FC) of DDX3X-derived iCLIP read abundance normalised to the RNA-seq read abundance of the corresponding tRNA isoacceptors in HEK293 cells. Individual rows at each tRNA isoacceptor level represent the FC values of corresponding tRNA-derived reads among all mapped tRNA sequences in replicate iCLIP experiments. (**F**) Representative EMSA after combining increasing molarities of MBP-DDX3X and 5′ end-labelled tRNA-Lys^UUU/CUU^ (10 nM final). UV-crosslinked RNPs were separated using nPAGE. Black arrowhead, non-bound tRNAs; red arrowhead, DDX3X-tRNA-Lys^UUU/CUU^ complexes. (**G**) Representative EMSA after combining increasing molarities of MBP-DDX3X and synthetic 5′ end-labelled tRNA-Lys^UUU^ sequences (30 nM final). UV-crosslinked RNPs were separated using nPAGE. Black arrowhead, non-bound tRNAs; red arrowhead, DDX3X-tRNA-Lys^UUU^ complexes. (**H**) Quantification of independent EMSA experiments (*n* = 3) for the calculation of the equilibrium dissociation constant (*K*_d_) between MBP-DDX3X and endogenously purified and 5′ end-labelled tRNA-Lys^UUU/CUU^ as well as a synthetic 5′ end-labelled tRNA-Lys^UUU^ sequence. Line marks the fit of the mean values (percent of shifted tRNA-Lys signal) to the hyperbolic binding isotherm used to calculate the *Kd* value while error bars represent standard deviations.

DDX3X, DDX5 and eIF4A1 are RNA helicases involved in many cellular processes (67,68). Although none of these RNA helicases have been implicated in mammalian tRNA or tsRNA biology, we focused only on DDX3X for further molecular characterization since this RNA helicase was detected by all employed enrichment approaches, and because DDX3X is thought to act on rather complex RNA structures in various RNA species (reviewed in (68)). To validate that DDX3X directly binds to tRNA-derived sequences, electrophoretic-mobility shift assays (EMSA) were performed using 5′ end-labelled and refolded tRNAs. Using increasing enzyme molarities revealed that MBP-DDX3X shifted a portion of the tRNA pool, indicating MBP-DDX3X bound to specific tRNAs (Supplementary Figure S4D). In contrast, eIF4A1-GST, which also showed buffer composition-dependent activity on 3′-tracer tRNAs, did not bind to tRNAs in its canonical buffer and at protein concentrations equimolar to MBP-DDX3X (Supplementary Figure S4D). To determine if DDX3X interacted with tRNAs *in vivo*, published data obtained from individual nucleotide cross-linking and immunoprecipitation (iCLIP) experiments in HEK293 cells ectopically expressing DDX3X-FLAG (69) were re-analysed using a newly developed non-coding RNA (ncRNA) ‘aware’ mapping tool. This tool performs sequential read mapping to various categories of abundant repetitive ncRNAs resulting in more accurate quantification of iCLIP-mediated protein binding to ncRNAs, including tRNAs, especially since such reads are often underrepresented when using standard mapping pipelines due to missing annotations and unresolved multimapping issues. The analysis revealed that between 10–20% of all iCLIP-derived reads mapped to the human tRNA sequence space (Figure 5A) suggesting that DDX3X interacted directly with tRNAs *in vivo*. Notably, As[III] exposure resulted in slightly increased iCLIP signals on tRNAs, which was not observed in cells overexpressing a DDX3X helicase domain mutant (DDX3X_R534H_, Figure 5B) suggesting a contribution of the helicase domain to DDX3X-tRNA interactions during the response to As[III]. Mapping individual cDNA read start positions (indicative of reverse transcription blocks by cross-links) to tRNA sequences showed cross-linking signatures at AC- and T-loop regions suggesting that DDX3X preferentially interacted with tRNAs through 3′ moieties (Figure 5C). Importantly, the analysis also revealed that DDX3X binding to tRNAs *in vivo* exhibited specificity towards particular tRNA isoacceptors (Figure 5D). This specificity was not a function of tRNA expression since normalising the abundance of tRNA-derived reads to their expression (based on matched total RNAseq datasets (69)) revealed no correlation between DDX3X binding and tRNA expression, therefore suggesting binding specificity of DDX3X towards particular tRNAs (Figure 5E). To validate the binding of DDX3X to specific tRNAs, tRNA-Lys^UUU/CUU^, which was among the top tRNA-derived iCLIP hits was affinity-purified from mammalian cells, ^32^P-labelled at the 5′ end, refolded and subjected to EMSA using MBP-DDX3X. The results showed that MBP-DDX3X bound purified endogenous tRNA-Lys^UUU/CUU^ quantitatively and by forming a single RNA-protein complex (Figure 5F and Supplementary Figure S4E) with a dissociation constant (*K*_d_) of 83.4 ± 47 nM (Figure 5H and Supplementary Table S4). As controls, EMSA were performed using synthetic tRNA-Lys^UUU^ (Figure 5G), which showed that MBP-DDX3X formed various RNA-protein complexes with a fraction of synthetic tRNA-Lys^UUU^ and a *K*_d_ of 431 ± 295 nM (Figure 5H and Supplementary Table S4). These results suggested that the binding of MBP-DDX3X to specific tRNAs was likely governed by correct RNA structures that are supported by particular RNA modifications, which were absent in synthetic tRNA sequences.

### DDX3X displays unwinding activity on structured 3‘-tracer tRNAs

**Figure 6.**
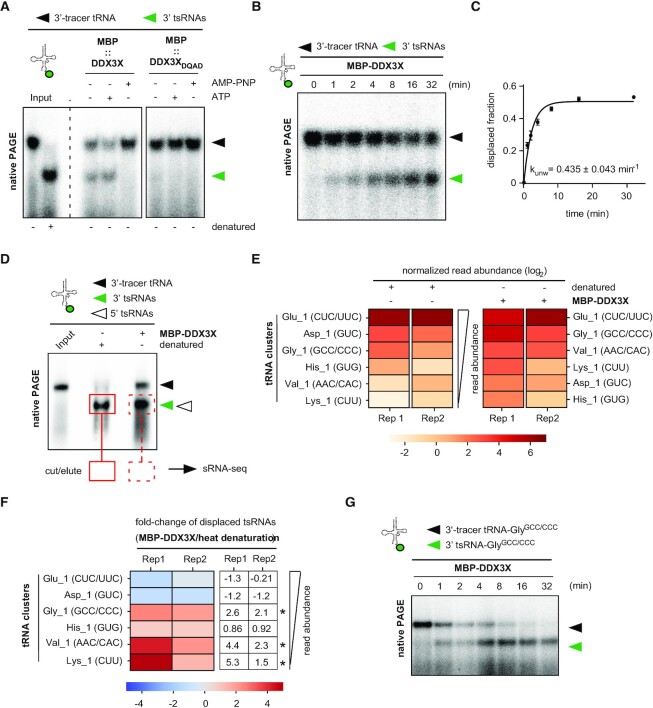
DDX3X separates 3′-tracer tRNAs with specificity for particular tRNA isoacceptors (**A**) Representative RNA helicase activity assay (fixed time-point) using equal molarities of recombinant MBP-DDX3X or MBP-DDX3X_DQAD_ (750 nM) and 3′-tracer tRNAs (60 nM final) in the presence of equimolar ATP/MgCl_2_ or AMP-PNP/MgCl_2_ (2 mM). Black arrowhead, 3′-tracer tRNAs; green arrowhead, 3′ tsRNAs. (**B**) Representative RNA helicase activity assay using MBP-DDX3X (750 nM) and 3′-tracer tRNAs (20 nM final) in the presence of equimolar ATP/MgCl_2_ (2 mM). Aliquots were removed from reactions at indicated time points and separated using nPAGE. Arrowheads, as described in (A). (**C**) Quantification of triplicate time-course RNA helicase activity assays (see Supplemental Figure S5B) using MBP-DDX3X (750 nM) and 3′-tracer tRNAs (10 nM final) in the presence of equimolar ATP/MgCl_2_ (2 mM) to derive *K_unw_*. Line marks the fit of the mean values to the integrated first-order rate equation, while error bars represent standard deviations. (**D**) Representative fixed-time point RNA helicase assay using MBP-DDX3X (750 nM) on 3′-tracer tRNAs (60 nM final) in the presence of equimolar ATP/MgCl_2_ (2 mM). 3′-tracer tRNAs that were heat-denatured were loaded in parallel. RNAs migrating at the level of heat-denatured and MBP-DDX3X-displaced 3′ tsRNAs (dashed and red squares, respectively) were excised in technical duplicates from nPAGE, cloned and subjected to small RNA sequencing. Black arrowhead, 3′-tracer tRNAs; green arrowhead, 3′ tsRNAs; white arrowhead, position where also non-labelled 5′ tsRNAs will migrate. (**E**) Heat maps representing relative read abundance (normalised log_2_ values) of known ANG tRNA substrates. The data was obtained by mapping replicate sRNA-seq data (columns) originating from heat-denatured 3′-tracer tRNAs (left panel) and MPB-DDX3X-displaced 3′-tsRNAs (right panel) to collapsed tRNA clusters as described in (52). Panels are ranked by relative abundance of the respective tRNA cluster in a descending manner. (**F**) Heat maps representing fold-changes of the relative read abundance (per tRNA cluster) after mapping reads originating from MPB-DDX3X-displaced tsRNAs divided by read abundance from heat-denatured 3′-tracer tRNAs (as described in (E)). Combined replicates (columns) ranked by relative abundance of the respective tRNA cluster in a descending manner. Asteriks denote tRNA isoacceptors with positive fold change ratios, indicating preferential activity of MBP-DDX3X on these tRNAs, which is independent of the corresponding tracer tRNA abundance in the 3′-tracer tRNA pool. (**G**) Representative time-course RNA helicase activity assay using MBP-DDX3X (750 nM) and 3′-tracer tRNA-Gly^GCC/CCC^ (10 nM final) in the presence of equimolar ATP/MgCl_2_ (2 mM). Aliquots were removed from reactions at indicated time points and separated using nPAGE. Arrowheads, as described in (A).

To validate that DDX3X directly binds tRNAs containing ANG-mediated ‘nicks’, 3‘-tracer tRNAs and MBP-DDX3X were incubated in its canonical buffer and analysed by EMSA. To minimise the release of RNA helicase substrate, the non-hydrolysable ATP analogue AMP-PNP was used. The results showed that MBP-DDX3X could directly associate with a fraction of 3‘-tracer tRNAs (Supplementary Figure S5A). Notably, eIF4A1-GST, which also showed activity on 3′-tracer tRNAs (albeit in non-canonical buffers), did not stably bind 3′-tracer tRNAs in its canonical buffer (Supplementary Figure S5A). Furthermore, denaturation of 3‘-tracer tRNAs prior to EMSA did not result in RNP formation indicating that MBP-DDX3X interacted with structured 3‘-tracer tRNAs but not individual 3′ tsRNAs (Supplementary Figure S5A). To address if DDX3X displayed ATP-dependent helicase activity towards 3‘-tracer tRNAs, *in vitro* activity assays were performed in the presence or absence of ATP. The results showed MBP-DDX3X-mediated separation of 3′ from 5′ tsRNAs occurred also independently of additional ATP (Figure 6A) confirming previous observations. To remove potentially contaminating ATP from the MBP-DDX3X preparation, MBP-DDX3X was pre-incubated with AMP-PNP to displace ATP, which clearly abrogated DDX3X activity on 3′-tracer tRNAs (Figure 6A). In addition, an ATPase-deficient version of recombinant DDX3X (MBP-DDX3X_DQAD_) displayed no activity on 3′-tracer tRNAs (Figure 6A), confirming that MBP-DDX3X displaced 3′ tsRNAs from 3‘-tracer tRNAs in an ATP-dependent fashion. Replicate single-turnover time-course activity assays, performed under pre-steady state conditions, revealed that MBP-DDX3X was only active on a fraction of 3‘-tracer tRNAs (Figure 6B and Supplementary Figure S5B) with an observed ‘unwinding’ constant (*k_unw_*) of 0.4347 ± 0.04352 min^−1^ and an ‘unwinding’ amplitude of 49.82 ± 0.01% (Figure 6C and Supplementary Table S4). Because ‘unwinding’ amplitudes of RNA helicases could be the result of concurrent RNA duplex separation and RNA annealing (70), MBP-DDX3X was also incubated with denatured 3‘-tracer tRNAs in the absence of ATP. Analysis for potential annealing activity using single-turnover kinetic assays followed by nPAGE indicated none to very weak MBP-DDX3X-mediated formation of RNA duplexes (Supplementary Figure S5C). These results indicated that MBP-DDX3X exhibited distinctively greater separation activity on 3‘-tracer tRNAs than annealing activity on denatured 3′-tracer tRNAs under these experimental conditions.

### Evolutionary conservation of DDX3X activity on ANG-processed tRNAs

DDX3X genes are evolutionary conserved (71). To determine if DDX3X activity on tRNAs containing ANG-mediated ‘nicks’ is conserved, belle, the *Drosophila melanogaster* homologue of human DDX3X (72) was purified as a fusion protein (MBP-belle) and tested for ATPase activity. MBP-belle hydrolysed ATP in the presence of RNA (Supplementary Figure S5D) indicating the purification of active enzyme. MBP-belle was incubated with 3′-tracer tRNAs, which were produced by incubating purified tRNA from *Drosophila* embryos with rANG, which had previously been shown to process tRNAs expressed by *Drosophila* cells (73). These 3′-tracer tRNAs were incubated with MBP-belle in different reaction buffers and MBP-belle activity was analysed by nPAGE. The results showed the separation of 3′ tsRNAs from a fraction of 3′-tracer tRNAs under particular reaction buffer conditions by MBP-belle (Supplementary Figure S5E). Using these buffer conditions, replicate single-turnover time-course activity assays were performed at pre-steady state conditions resulting in a *k*_unw_ of 0.934 ± 0.176 min^−1^ and an ‘unwinding’ amplitude of 0.36 ± 0.014% (Supplementary Figure S5F and Supplementary Table S4). These results confirmed that belle, the fruit fly homologue of mammalian DDX3X, was able to bind and separate 3′-tracer tRNAs *in vitro*.

### DDX3X activity displays specificity towards particular ANG-substrate tRNAs

Since the activity of MBP-DDX3X affected only a fraction of 3‘-tracer tRNAs, fixed-time point activity assays were performed, followed by gel purification of RNAs migrating at the level of displaced 3′ tsRNAs. As controls, tsRNAs that had been displaced by denaturation were eluted (Figure 6D). sRNAseq was performed to reveal the identities of tsRNAs that were produced by rANG followed by heat denaturation (all potential RNA helicase substrates) versus those tsRNAs displaced by MBP-DDX3X activity only. Using the ranking of the most abundant tRNA-derived reads after rANG incubation (Supplementary Figure S1D) as basis and plotting the relative abundance of tsRNA-derived reads resulting from heat denaturation versus MBP-DDX3X-mediated activity on 3‘-tracer tRNAs suggested that MBP-DDX3X separated 3′-tracer tRNAs not strictly based on tRNA abundance in the 3′-tracer tRNA pool (Figure 6E). Notably, comparing the relative abundance of tsRNA-derived reads resulting from MBP-DDX3X-mediated activity on 3‘-tracer tRNAs with those obtained after denaturation of 3‘-tracer tRNAs showed that a positive fold-change was only detectable for a subset of tRNAs (tRNA-Lys^CUU^, tRNA-Val^AAC/CAC^, tRNA-Gly^GCC/CCC^, Figure 6F). These observations suggested that particular tRNA identities represented preferential substrates for DDX3X-mediated activity on 3′-tracer tRNAs. To test this directly, tRNA-Gly^GCC/CCC^ was affinity-purified from human cells, 5′-labelled or processed to obtain 3‘-tracer tRNA-Gly^GCC/CCC^, both of which were subjected to EMSA using MBP-DDX3X. The results showed that both tRNA-Gly^GCC/CCC^ as well as 3‘-tracer tRNA-Gly^GCC/CCC^ could be shifted to similarly sized RNPs, which indicated that MBP-DDX3X interacted with both tRNA-Gly^GCC/CCC^ preparations (Supplementary Figure S6A). Notably, single-turnover time-course activity assays performed under pre-steady state conditions revealed that MBP-DDX3X quantitatively separated 3′ tsRNAs from 3‘-tracer tRNA-Gly^GCC/CCC^ (Figure 6G). These results confirmed that MBP-DDX3X displayed specificity towards particular isoacceptors within the pool of 3‘-tracer tRNAs, suggesting (unknown) denominators determining DDX3X activity.

### Specific RNA modifications affect RNA structure but not DDX3X activity on 3′-tracer tRNAs

It is currently assumed that DEAD box RNA helicases recognize RNAs through structural elements with poorly defined sequence context (74). In support of this notion, CLIP-based approaches have not produced testable consensus sequence motifs for DDX3X binding to RNAs, albeit complex secondary structures and high GC content have been suggested as targets of DDX3X binding and activity (69,75,76). To address if RNA modifications could affect DDX3X activity towards specific 3′-tracer tRNAs, including 3‘-tracer tRNA-Gly^GCC/CCC^, we aimed at modulating the RNA modification status of 3′-tracer tRNAs. As an unmodified tRNA sequence, a synthetic mimic of tRNA-Gly^GCC^ harbouring no modifications was ^32^P-labelled at the 5′-end, folded in the presence of Mg^2+^ and subjected to rANG-mediated hydrolysis. nPAGE analysis of this 5′-tracer tRNA sequence after denaturation revealed no 5′ tsRNAs with higher mobility suggesting that rANG was unable to efficiently hydrolyse synthetic tRNA-Gly^GCC^ sequences (Supplementary Figure S6B). This indicated that in the absence of RNA modifications the formation of the correct AC-loop structure, a prerequisite for ANG activity, was largely impaired. Next, purified tRNAs from HAP1 cells harbouring a mutation in the TRMT10A gene were used for the production of 3′-tracer tRNAs. This gene encodes a guanosine-specific tRNA methyltransferase that targets position 9 (m^1^G9) of a subset of nuclear encoded tRNAs (77), which is potentially involved in suppressing the production of a specific 5′ tsRNA in specific cell types (78). tRNAs from *TRMT10A* mutant cells were efficiently hydrolysed by rANG and could be maintained as structured 3′-tracter tRNAs during nPAGE (Supplementary Figure S6C). Replicate single-turnover time-course activity assays under pre-steady state conditions were used to calculate *k*_unw_ and ‘unwinding’ amplitudes for MBP-DDX3X activity on 3′-tracer tRNAs extracted from controls and *TRMT10A* mutant cells (Supplementary Figure S6D, E and Supplementary Table S4). The results indicated that the absence of m^1^G9 from a subset of cytoplasmic tRNAs did not modulate the efficiency of MBP-DDX3X to separate 3′-tracer tRNAs *in vitro* in a statistically significant manner (p-value 0.7698, two-tailed and unpaired t-test). To remove more than one RNA modification from individual 3′-tracer tRNAs, tRNAs extracted from HeLa cells were treated with recombinantly expressed AlkB (rAlkB), an enzyme with dealkylating activity for specific RNA modifications (i.e. m^1^A, m^1^G, m^2^_2_G and m^3^C (30)), which can be found in tRNAs that are targeted by ANG (79). Two rAlkB forms (AlkB and AlkB_D135S_) were activity-tested using synthetic RNA oligos carrying m^1^A or m^1^G at their 5′ ends. The result confirmed robust demethylation activity of both oligos in a rAlkB concentration-dependent manner (Supplementary Figure S6F). Notably, a fraction of 3′-tracer tRNAs produced from AlkB-treated tRNAs was unstable during nPAGE indicating the requirement of particular RNA modifications for the structural integrity of some tRNAs containing ANG-mediated ‘nicks’ (Supplementary Figure S6G). Replicate single-turnover time-course activity assays under pre-steady state conditions were performed to test if the remaining AlkB-treated and structurally maintained 3′-tracer tRNAs would be processed differently than controls by MBP-DDX3X. The results showed that AlkB-treated and structurally maintained 3′-tracer tRNAs were separated by MBP-DDX3X with similar unwinding constants and amplitudes as controls, the difference of which was not statistically significant (*P*-value 0.3161, two-tailed and unpaired t-test, Supplementary Figure S6H, I and Supplementary Table S4) suggesting that AlkB-sensitive RNA modifications affected likely 3′ tracer tRNA structure and, therefore, only indirectly the activity of MBP-DDX3X on 3′ tracer tRNAs.

### Combined DDX3X and DDX5 depletion does not abrogate activities on 3‘-tracer tRNAs *in vitro*

**Figure 7.**
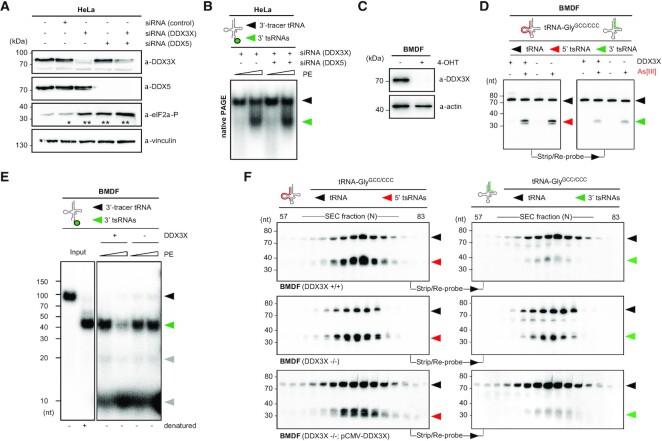
DDX3X affects the stability of 3′-tracer tRNAs *in vitro* and *in vivo*. (**A**) Western blotting for DDX3X, DDX5 and eIF2α (serine-51 phospho) before and 48 h after transfection with siRNA pools (non-targeting as controls versus targeting each RNA helicase alone or in combination). Antibodies against vinculin were used as a loading control. Lane marked with (*) points at background eIF2α-P signal, while lanes marked with (**) indicate siRNA-mediated induction of eIF2α-P signal in RNA helicase knockdowns. (**B**) 3′-tracer tRNAs (8 nM final) were incubated with cytoplasmic protein extracts (2 or 4 μg) obtained from HeLa cells transfected with siRNAs targeting DDX3X or DDX3X in combination with DDX5. Reactions were separated by nPAGE and 3′ tsRNA signals were collected as described above. Black arrowhead, 3′-tracer tRNAs; green arrowhead, 3′ tsRNAs. (**C**) Western blotting for DDX3X in total protein extract obtained from BMDF after 72 h of 4-hydroxytamoxifen (4-OHT)-mediated deletion of the DDX3X gene locus. β-actin was used as a loading control. (**D**) NB using sequential probing of the same membrane against the 5′ and 3′ portions of tRNA-Gly^GCC/CCC^ on total RNA purified from BMDF after 72 h of 4-OHT-mediated deletion of the DDX3X gene locus before and after As[III] exposure (0.5 mM for 1 h). Black arrowheads, mature tRNAs; red arrowhead, 5′ tsRNAs; green arrowhead, 3′ tsRNAs. (**E**) 3′-tracer tRNAs (8 nM final) were incubated with cytoplasmic protein extracts (5 or 10 μg) obtained from BMDF before or after 4-OHT-mediated deletion of the DDX3X. Reactions were separated by nPAGE and 3′ tsRNA signals were collected as described above. Black arrowhead, 3′-tracer tRNAs; green arrowhead, 3′ tsRNAs; grey arrowhead, signals from partial 3′ tsRNA degradation. (**F**) NB of total RNA purified from SEC fractions originating from As[III]-exposed BMDF before (upper panels), after 4-hydroxytamoxifen (4-OHT) mediated deletion of DDX3X (middle panels), and in the presence of ectopic expression of wild type DDX3X (pCMV-DDX3X, lower panels) using sequential probing of the same membrane. Probes for the 5′ (left panels) and 3′ (right panels) moieties of tRNA-Gly^GCC/CCC^ were used. Black arrowheads, mature tRNAs; red arrowhead, 5′ tsRNAs; green arrowhead, 3′ tsRNAs.

To address if a loss-of-function of DDX3X might affect tRNA and/or tsRNA levels and/or integrity, especially during the response to As[III], we used siRNA-mediated knockdown (KD) of DDX3X and/or DDX5 in HeLa cells. Western blotting confirmed the downregulation of both enzymes albeit traces of DDX3X were still detectable after KD (Figure 7A). Notably, probing for the phosphorylated form of eIF2α indicated the activation of stress responses after siRNA-mediated KD of either or both RNA helicases suggesting that impairing DDX3 or DDX5 function resulted in cellular stress. NB showed that reduced expression of DDX3X and/or DDX5 had no apparent effect on the steady-state levels of tRNAs before and after exposure to As[III] as determined by probing for tRNA-Gly^GCC/CCC^ (Supplementary Figure S7A). An apparent increase in 5′ and 3′ tsRNA levels after As[III]-induced stress was not specific to either DDX3X and/or DDX5 dose reduction since also control siRNAs caused similar changes in tsRNA signals (Supplementary Figure S7A). These observations suggested that transfection of siRNAs activated cellular pathways resulting in tRNA fragmentation that was independent of eIF2α as had been observed before for stress-induced tRNA fragmentation (31,80). Similar observations were made after KD of DDX3X in HeLa or HEK293 cells using virus-mediated expression of short-hairpin RNAs (shRNAs) (Supplementary Figure S7B, C), which confirmed that downregulation of DDX3X expression did neither affect mature tRNA-Gly^GCC/CCC^ levels nor the levels of tsRNAs derived from these tRNA isoacceptors while virus infection or short hairpin RNA expression increased tsRNA levels. Furthermore, incubation of cytoplasmic protein extracts (after siRNA KD) with 3′-tracer tRNAs still revealed separation of 3′ tsRNAs indicating that either remaining traces of DDX3X, DDX5 or additional RNA helicases (i.e. eIF4A1) remained active in KD extracts (Figure 7B). These combined results indicated that manipulation of RNA helicase levels by siRNA-mediated KD did not allow deciphering the potential contribution of multi-functional and ubiquitous RNA helicases such as DDX3X or DDX5 to tRNA and tsRNA levels or integrity, especially during the response to oxidative stress.

### DDX3X depletion does not abrogate separation but affects the stability of 3‘-tracer tRNAs *in vitro*

To remove DDX3X activity quantitatively from cells, we used mouse bone marrow-derived fibroblasts (BMDF) containing a genetically engineered DDX3X locus, which allows recombination-mediated excision of the DDX3X locus from the genome through addition of 4-hydroxytamoxifen (4-OHT) to cultured cells (81). Deletion of the DDX3X locus reduced DDX3X expression to almost undetectable protein levels by 72 h of continuous 4-OHT treatment (Figure 7C). NB for tRNA-Gly^GCC/CCC^ before and after exposure to As[III] confirmed that loss of DDX3X function had no effect on tRNA or tsRNA levels (Figure 7D). To address if DDX3X depletion from cells affected the separation and degradation of 3′-tracer tRNAs *in vitro*, cytoplasmic protein extracts from DDX3X-depleted BMDFs were incubated with 3‘-tracer tRNAs followed by nPAGE analysis. The results showed displacement of 3′ tsRNAs from 3‘-tracer tRNAs in DDX3X-depleted protein extracts, although higher protein concentrations were required than when using DDX3X-containing protein extracts (Supplementary Figure S7D), which confirmed redundancy through other activities such as DDX5 and/or eIF4A1 but indicated that DDX3X in cytoplasmic protein extracts played a potentially major role in separating 3‘-tracer tRNAs *in vitro*. Notably, 3′ tsRNA-degradation, seen in particular buffer conditions, appeared to be DDX3X-dependent since increasing amounts of DDX3X-containing extracts displayed a protein concentration-dependent loss of 3′ tsRNAs, while increasing amounts of DDX3X-depleted cytoplasmic protein extracts maintained equal 3′ tsRNA levels (Figure 7E). These observations suggested that DDX3X activity on 3′-tracer tRNAs was a prerequisite for the access of 3′ tsRNA-degrading activities *in vitro*.

### DDX3X dose affects 3′ tsRNA stability *in vivo*

To test for a role of DDX3X on the biogenesis and stability of stress-induced tsRNAs *in vivo*, BMDF (with or without deleted DDX3X locus) were exposed to As[III], followed by cell lysis, SEC fractionation and NB for tRNA-Gly^GCC/CCC^. This revealed that fractionated RNPs from BMDF expressing endogenous DDX3X (controls) contained relatively fewer 3′ tsRNAs-Gly^GCC/CCC^ than 5′ tsRNAs-Gly^GCC/CCC^ in, which confirmed 5′/3′ tsRNA asymmetry *in vivo* (Figure 7F). In contrast, RNPs extracted from DDX3X-depleted BMDF contained almost equal levels of 5′ and 3′ tsRNAs-Gly^GCC/CCC^ (Figure 7F) indicating stabilization of 3′ tsRNAs in the absence of DDX3X. These observations suggested that DDX3X function could be required for the degradation of 3′-tsRNAs after ANG-mediated tRNA hydrolysis. Notably, transfection of a CMV-promoter containing plasmid harbouring the coding sequence of DDX3X into DDX3X-depleted BMDF resulted in re-establishment of the apparent 5′/3′ tsRNA asymmetry (Supplementary Figure S7E, F and Figure 7F). These combined results indicated a contributing role for DDX3X to 5′ tsRNA biogenesis *in vivo*, specifically by promoting the instability of 3′ tsRNAs after ANG-mediated hydrolysis and separation of 'nicked' tRNA isoacceptors.

## DISCUSSION

tsRNAs have attracted increased interest over the last decade, not only because of their proposed connection to various biological processes, but also due to their association with a multitude of pathological conditions, especially in humans (reviewed in (27)). Importantly, the physiological impact of tsRNAs has largely been attributed to individual tsRNA sequences, likely through their capability of interacting with other RNAs and proteins (8,13,21,32,53,82). In addition, intermolecular interactions between tsRNAs through G-quadruplexes (66) or by homo-or heterotypic dimerization of specific 5′ tsRNAs have been reported (83). If individual tsRNAs convey molecular function and physiological impact, then a major unresolved question relates to how one endonuclease-mediated tRNA hydrolysis event resulting in ‘nicked’ tRNAs would give rise to individual tsRNAs capable of interacting with other molecules. This is likely not a spontaneous process governed by thermodynamics and stochasticity but would require cellular activities capable of separating and/or annealing tRNA-derived sequences. Intriguingly, the molecular identity of such activities still remains unknown. Assuming that such activities exist, we established an *in vitro* assay capable of detecting enzymatic activities which can bind and process tRNAs with endonuclease-mediated ‘nicks’ (tracer tRNAs). This so-called ‘tracer tRNA’ assay is experimentally versatile since it can be modulated at the level of substrate tRNA-derived sequences (*i.e*. synthetic, endogenously modified, isoacceptor-purified, structured RNAs) and at the source of the catalytic activities to be tested (*i.e*. recombinant proteins, protein extracts depleted of candidate activities). Using this assay in combination with protein lysates from various mammalian cell lines indicated the presence of enzymatic activities that can separate ‘nicked’ tRNAs, thereby producing individual 5′ and 3′ tsRNAs, as well as of nucleolytic activities, which partially degraded 3′ tsRNA moieties, particularly from their 3′ ends. To identify these activities, we reasoned that they should be in close proximity to tRNAs, ‘nicked’ tRNAs and, potentially also tsRNAs, particularly during the stress response *in vivo*. By tracking RNPs after As[III]-induced tRNA fragmentation using biochemical fractionation in human cells, we identified many proteins that co-migrated with both tRNAs and 5′ tsRNAs during the acute stress response. Among these, various proteins stood out for having the potential to contribute to tsRNA biogenesis. Notably, we identified RNaseT2, but not ANG, as co-migrating with tsRNAs-containing RNPs during the response to oxidative stress. RNaseT2 is responsible for tsRNA production in *Saccharomyces cerevisiae* (84), *Tetrahymena* (85) and *Arabidopsis* (86). Importantly, recent reports showed that ANG-deficient cells still produced stress-inducible tsRNAs (23,24), suggesting the existence of redundant nuclease activities acting on tRNAs during the stress response. However, even though human RNaseT2 hydrolysed tRNAs *in vitro* (86), its activity on tRNAs in human cells *in vivo* remains to be experimentally tested. Furthermore, in light of the observed partial *in vitro* degradation of 3′ tracer tRNAs by unknown activities contained in cytoplasmic extracts, it remains to be tested if RNAseT2 could be one of the responsible enzymes mediating this activity on ‘nicked’ tRNAs *in vitro* as well as *in vivo*.

Stress-induced tsRNAs-containing RNPs were also associated with DEAD box RNA helicases suggesting that these enzymes could contribute to tsRNA biogenesis by producing individual 5′ and 3′ tsRNAs. Importantly, among those tested RNA helicases that localized to SG during the As[III]-induced stress response, we identified three RNA helicases (eIF4A1, DDX3X and DDX5), which were able to accept and robustly separate ‘tracer tRNAs’ under specific *in vitro* reaction conditions. eIF4A1, DDX3X and DDX5 are ubiquitously expressed enzymes involved in many cellular processes including stress responses (63,67,87,88). All three enzymes localize to human SG in high copy numbers (63) and our co-immunoprecipitation data from SG core-enriched material as well as immunofluorescence data support that eIF4A1, DDX3X and DDX5 can form protein complexes *in vivo*, especially during the acute stress response.

Do these RNA helicases also interact with tRNA-derived sequences? Generally, the determinants underlying RNA substrate specificities for specific DEAD box RNA helicases remain ill-defined (89). *In vitro* studies using dsRNA substrates indicated that DDX3X and DDX5 unwound short double stranded RNA substrates with 3′ single stranded overhangs (71,90–94), and the available *in vivo* data suggested no requirement for consensus sequences (69,75,76). Similarly, eIF4A, considered to be the ‘godfather’ of DEAD box helicases (95), appears to have no defined substrate preference nor consensus sequence requirement except that unstructured RNAs of a certain length activate its ATPase activity *in vitro* (96,97). Importantly, most enzymatic studies on DEAD box RNA helicases have been performed using synthetic dsRNA substrates, which can be called biologically irrelevant RNA substrates. Notable exceptions are the use of synthetic segments of HIV-1 RNA as substrates for DDX3X (37) and human miRNA precursors as substrates for DDX5 (98). Importantly, DEAD box RNA family members such as human DDX1 or the yeast homologue of DDX5 (DBP2) have been implicated in tRNA biology, albeit at the level of tRNA splicing (64,65), whereas human DHX36 was reported to bind to 5′ tsRNAs that have the propensity of creating RNA-G quadruplex structures (66). Beside these observations, direct biochemical activity by DEAD box RNA helicases on tRNAs or tRNA-derived sequences has, so far, not been reported. In this respect, our data are the first reporting on activities of eIF4A1, DDX3X and DDX5 towards endogenous, post-transcriptionally modified and structured tRNA-derived sequences.

Are these interactions occurring *in vivo* and are they biologically relevant? We focused on DDX3X for a more detailed characterization of its tRNA interactions. DDX3X binding to RNA substrates has been approached before by using iCLIP methodology, which revealed interactions mostly with coding RNAs, but also with 18S rRNA (69,75,76). Notably, re-analysis of FAST-iCLIP data sets (69) using a newly established mapping pipeline, and omitting exclusion of tRNA-derived and other multimapping reads, indicated that overexpressed DDX3X preferentially interacted with sequences derived from the 3′ portions of a subset of tRNA isoacceptors *in vivo*. These interactions appeared to be independent of tRNA isoacceptor expression levels in HEK293 cells, and both the separation of only a fraction of the 3′-tracer tRNA pool into 5′ and 3′ tsRNAs as well as the cloning of displaced tsRNAs from ‘tracer tRNA’ assays indicated specificity for DDX3X activity towards particular 3′-tracer tRNAs. However, this interpretation of RNA-seq data is currently technically biased since data from (69) as well as our data were not obtained from RNA input that was controlled for the potential influence of RT-affecting post-transcriptional modifications. Notably, we confirmed a degree of specificity for DDX3X-tRNA interactions when using endogenously expressed and natively purified tRNA-Gly^GCC/CCC^ for 3′-tracer tRNA production and as DDX3X substrate *in vitro*, which showed that this specific ‘tracer tRNA’ was quantitatively separated into 5′ and 3′ tsRNAs. In contrast, using synthetic tRNA-Gly^GCC^ for 3′-tracer tRNA production indicated that tRNA modifications were required for correct folding of tRNA sequences, without which recombinant ANG was unable to hydrolyse the AC-loop. In addition, *in vitro* removal of various RNA modifications from the purified tRNA pool before 3′-tracer tRNA production did not affect DDX3X activity on such 3′-tracer tRNAs but affected their structural integrity as visualized by nPAGE. These combined results revealed technical limitations of the ‘tracer tRNA’ approach, which prevent us from answering if specific RNA modifications could be determining DDX3X binding to and/or its activity on specific 3′-tracer tRNAs. However, the combined observations suggest that structural integrity of tRNA-derived sequences is one important denominator, first and foremost as a prerequisite for endonucleolytic hydrolysis by AC-loop nucleases, but subsequently also for access of ‘downstream’ activities to ‘nicked’ tRNAs, at least *in vitro*. Structural integrity of tRNAs supported by RNA modifications is a pre-requisite for correct selection by aminoacyl-tRNA synthetases (aaRS), a member of which has been reported to affect the levels of a particular stress-independent tsRNA species (3′ tsRNA-Leu^CAG^) in human cells with functions in ribosome biogenesis (13,32,99). Whether or not aaRS and their interactors prevent or allow access of RNA helicases, such as DDX3X, to ‘nicked’ tRNAs *in vivo* might only be conclusively answered by determining RNA helicase protein interactomes with subcellular resolution.

Could eIF4A1, DDX5 and DDX3X be involved in tsRNA biogenesis *in vivo*? Their ubiquitous expression in most cell types and their multifaceted roles in many cellular processes makes testing such a specific function, on a tiny fraction of cellular RNAs during specific (stress) conditions, among their many other roles experimentally very challenging. As for DDX3X, its deletion resulted in prenatal lethality in mice (100,101). In our experiments, neither knocking-down DDX3X expression by siRNAs or shRNAs, nor an inducible knock-out of DDX3X in cultured cells did affect the levels of specific tRNAs, or the levels of As[III]-induced tsRNAs, as determined by NB for specific tRNA isoacceptors. Furthermore, neither DDX3X KD alone or a combinatorial KD of DDX3X and DDX5 by siRNAs affected the separation of 3′-tracer tRNAs by cytoplasmic protein extracts *in vitro*, which supported the notion of redundancy in RNA helicase activities able to act on 3′-tracer tRNAs. However, 3′-tracer tRNA assays performed with protein extracts from DDX3X knockout cells revealed that DDX3X dose affected both the degree of tsRNA separation and, importantly, the stability of those 3′ tsRNAs that became separated from 3′-tracer tRNAs. This result indicated an interaction between DDX3X-mediated 3′-tracer tRNA separation and unknown RNA-degrading activities targeting the 3′ moiety of ‘tracer tRNAs’ for either 3′ to 5′ (exo-) or endonucleolytic degradation. Importantly, this observation was corroborated by probing SEC fractions from cells containing DDX3X, cells with a deleted DDX3X locus and those in which DDX3X expression was reconstituted by plasmid transfection, for tsRNA abundance, which revealed that DDX3X dose affected 3′ tsRNA levels *in vivo*. This is surprising given that probing tRNA integrity and tsRNA levels in total RNA pools during the response to As[III] by NB had not revealed differences in 3′ tsRNA levels between cells with or without DDX3X. One explanation allowing to reconcile these observations is the timing of RNA extraction from the two sample categories. While total RNA extraction from DDX3X KD and knockout cells using Trizol (category 1) immediately neutralizes cellular RNA degradation activities, production of protein lysates, their processing by gel filtration followed by RNA extraction from SEC fractions (category 2) is a rather elaborate process, which might have supported 3′ tsRNA-degrading activities in time. Of note, RNAse-inhibitor cocktail was present in the protein extraction buffers but not in the large volumes of gel filtration and elution buffers, which might have supported revealing such activities.

Taken together, the presented findings show that tRNAs containing ANG-mediated ‘nicks’ can be substrates for specific ATP-dependent RNA helicases, resulting in the separation of 5′ from 3′ tsRNAs *in vitro*. DDX3X binding to tRNAs *in vivo* and the effects of DDX3X deletion on 3′ tsRNA stability suggest that DDX3X plays a prominent role in contributing to the molecular processing of endonuclease-hydrolysed tRNAs into individual tsRNAs *in vivo*. The phenomenon of widespread tsRNA abundance in many species, the functional association of defined tsRNAs with specific cellular processes and the potential for specific tsRNAs to serve as biomarkers for human diseases, suggests that identifying the complete molecular machinery involved in producing tsRNAs is of great importance for our understanding as to how these small RNAs exert biological impact.

## Supplementary Material

gkad033_Supplemental_Files

## Data Availability

Small RNA sequencing data has been deposited at NCBI GEO database under accession number GSE187021. The mass spectrometry proteomics data have been deposited to the ProteomeXchange Consortium via the PRIDE (102) partner repository under accession number: PXD029869. The ncRNA-aware CLIP pipeline is available at the Zenodo repository (doi: 10.5281/zenodo.7524185). The DDX3X iCLIP data and matched RNA-Seq was previously published and is available from the Gene Expression Omnibus (GEO) GSE70804 (69).
