# Supplementary material for: Identification of RNA helicases with unwinding activity on angiogenin-processed tRNAs
Source: Nucleic Acids Res. 2023 Jan 31;51(3):1326–52. doi: 10.1093/nar/gkad033 (PMC9943664; doi:10.1093/nar/gkad033)
Supplement: gkad033_Supplemental_Files [file gkad033_supplemental_files.zip › Legends for Supp Tables_Drino.docx]

**Supplementary Table 1**: Summary of mass spectrometry data. Peptide identities and their corresponding intensities originating from tsRNAs-containing RNPs as well as control RNPs are shown. Relating to **Figure 2** and **Supplementary Figure 2**.

**Supplementary Table 2**: Gene Ontology annotation of protein identities identified as co-fractionating with tsRNAs-containing RNPs. Relating to **Supplementary Figure 2**.

**Supplementary Table 3**: List of known Stress Granule (SG)-associated proteins from previous reports, and their overlap with proteins identified to co-fractionate with tsRNAs-containing RNPs. Relating to **Supplementary Figure 3**.

**Supplementary Table 4**: Prism 5 values summary. The output of used mathematical functions and statistical tests to calculate unwinding and binding constants are listed. Relating to **Supplementary Figure 4, 5, 6** and **Figures 5, 6**.
