# Supplementary material for: Identification of RNA helicases with unwinding activity on angiogenin-processed tRNAs
Source: Nucleic Acids Res. 2023 Jan 31;51(3):1326–52. doi: 10.1093/nar/gkad033 (PMC9943664; doi:10.1093/nar/gkad033)
Supplement: gkad033_Supplemental_Files [file gkad033_supplemental_files.zip › Supplementary Figures+legends.pdf]

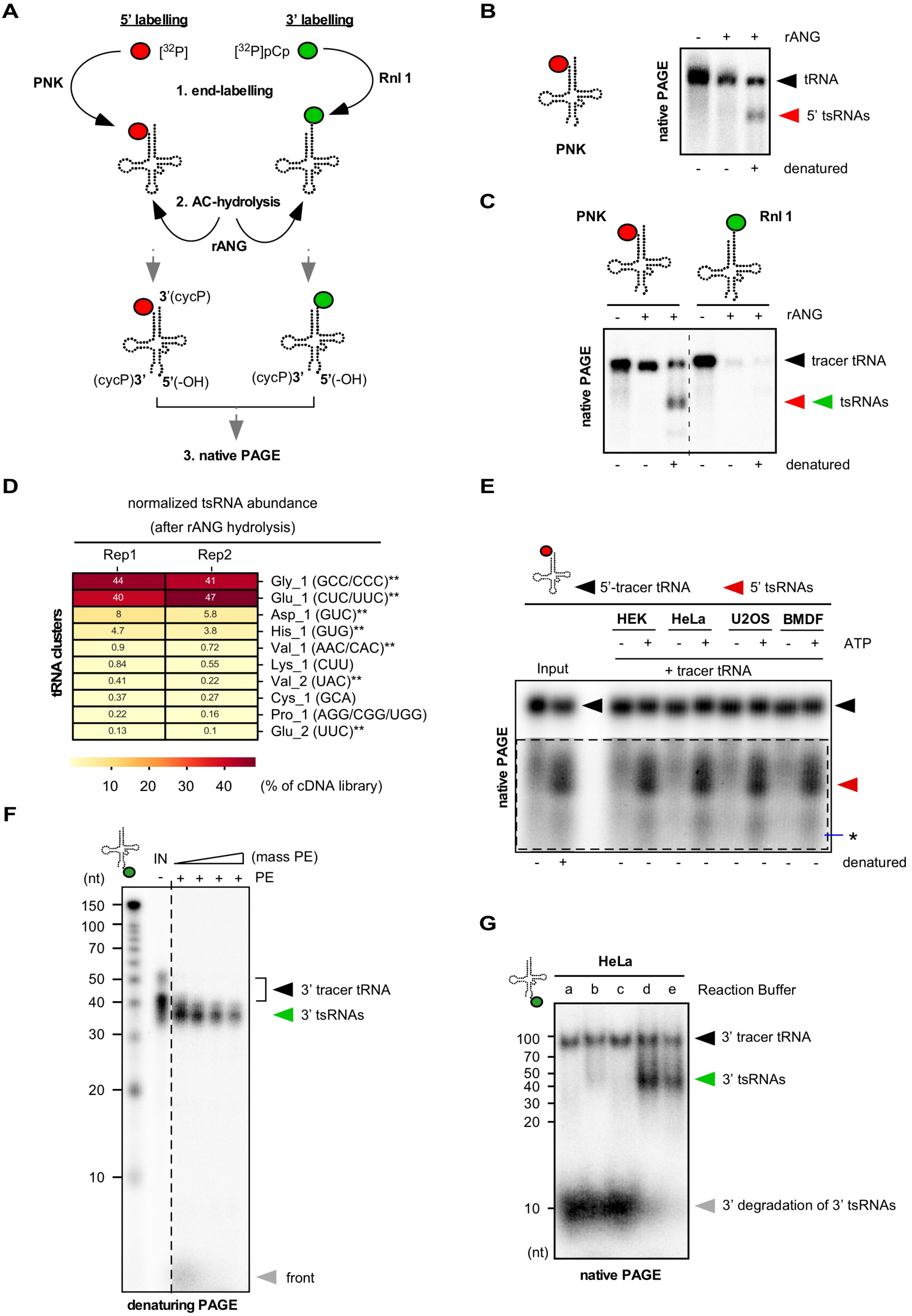
A
5' labelling
3' labelling
[32P]
[32P]pCp
PNK
Rnl 1
1. end-labelling
2. AC-hydrolysis
rANG
3'(cycP)
(cycP)3'
5'(-OH)
3. native PAGE
B
rANG
native PAGE
tRNA
5' tsRNAs
PNK
denatured
C
PNK
Rnl 1
rANG
native PAGE
tracer tRNA
tsRNAs
denatured
D
normalized tsRNA abundance
(after rANG hydrolysis)
Rep1
Rep2
tRNA clusters
Gly_1 (GCC/CCC)** 44 41
Glu_1 (CUC/UUC)** 40 47
Asp_1 (GUC)** 8 5.8
His_1 (GUG)** 4.7 3.8
Val_1 (AAC/CAC)** 0.9 0.72
Lys_1 (CUU) 0.84 0.55
Val_2 (UAC)** 0.41 0.22
Cys_1 (GCA) 0.37 0.27
Pro_1 (AGG/CGG/UGG) 0.22 0.16
Glu_2 (UUC)** 0.13 0.1
10 20 30 40 (% of cDNA library)
E
5'-tracer tRNA
5' tsRNAs
HEK
HeLa
U2OS
BMDF
ATP
Input
+ tracer tRNA
native PAGE
denatured
F
IN
(mass PE)
PE
(nt)
150 100 70 50 40 30 20 10
3' tracer tRNA
3' tsRNAs
front
denaturing PAGE
G
HeLa
a b c d e
Reaction Buffer
100 70 50 40 30 20 10
(nt)
3' tracer tRNA
3' tsRNAs
3' degradation of 3' tsRNAs
native PAGE

Supplementary Figure 1. Establishment of the 'tRNA tracer' assay

(**A**) Schematic representation of sequential enzymatic reactions for the production of 'tracer tRNAs' containing either labelled 5' or 3' tsRNA moieties. Total tRNA pools were either 5'-end labelled using ^{32}P-γ-ATP and T4 polynucleotide kinase (PNK), or 3' end-labelled using ^{32}P-cytidine 3', 5'-bisphosphate (pCp) and T4 RNA ligase 1 (Rnl), followed by treatment with recombinant ANG (rANG) and analysis by nPAGE. 5' position of label (^{32}P) is marked by a red dot; 3' position of label (^{32}P) is marked by a green dot; cycP, 2'-3' cyclic phosphate; -OH, hydroxyl moieties; AC-anticodon loop.

(**B**) Representative nPAGE of 5'-tracer tRNAs before and after heat denaturation. black arrowhead, tRNAs; red arrowhead, 5' tsRNAs.

(**C**) Representative nPAGE of 5'- and 3'-tracer tRNAs before and after heat denaturation; black arrowhead, ^{32}P-labelled 'tracer tRNAs'; red arrowhead, 5' tsRNAs; green arrowhead: 3' tsRNAs.

(**D**) Heat map representing relative abundance of tRNA-derived reads originating from denatured 3'-tracer tRNAs after mapping to particular tRNA isoacceptors (tRNA clusters, rows) as described in (40). Numbers represent the percentage of the most abundant 10 tRNA clusters (representing ≥ 0.1% of all tRNA-derived reads) per sequenced library (two technical replicates, columns). Asterisks denote ANG-substrate tRNAs as reported in (45, 47, 93).

(**E**) 5'-tracer tRNAs (8 nM final) were incubated with cytoplasmic protein extracts (2 µg) obtained from different cell lines in the presence or absence of 2 mM ATP. Reactions were separated by nPAGE and ^{32}P-signals were collected by exposing PA gels to phosphor-imaging plates for ≤ 2 hours. Black arrowhead, 5'-tracer tRNAs; red arrowhead, 5' tsRNAs; Asterisks denotes a digitally enhanced region of the image for better visualization of 5' tsRNA signals.

(**F**) 3'-tracer tRNAs (8 nM final) were incubated with increasing mass of cytoplasmic protein extracts (1, 2, 5, 10 µg) obtained from BMDF in the presence of 2 mM ATP. RNA was phenol-extracted from reactions and separated on denaturing PAGE followed as described above. Black arrowhead, 3'-tracer tRNAs; green arrowhead, 3' tsRNAs.

(**G**) 3'-tracer tRNAs (8 nM final) were incubated with cytoplasmic protein extracts (2 µg) obtained from HeLa cells and dialysed into 5 different reaction buffers (a-e) in the presence of 2 mM ATP. Different reaction buffers are as follows: a) DDX3X helicase buffer; b) common RNA helicase buffer; c) eIF4A1 helicase buffer; d) DDX1 helicase buffer; e) DDX5 helicase buffer). Reactions were separated by nPAGE and 3' tsRNA signals were collected as described above. Black arrowhead, 3'-tracer tRNAs; green arrowhead, 3' tsRNAs; grey arrowhead, signals from partial 3' tsRNA degradation.

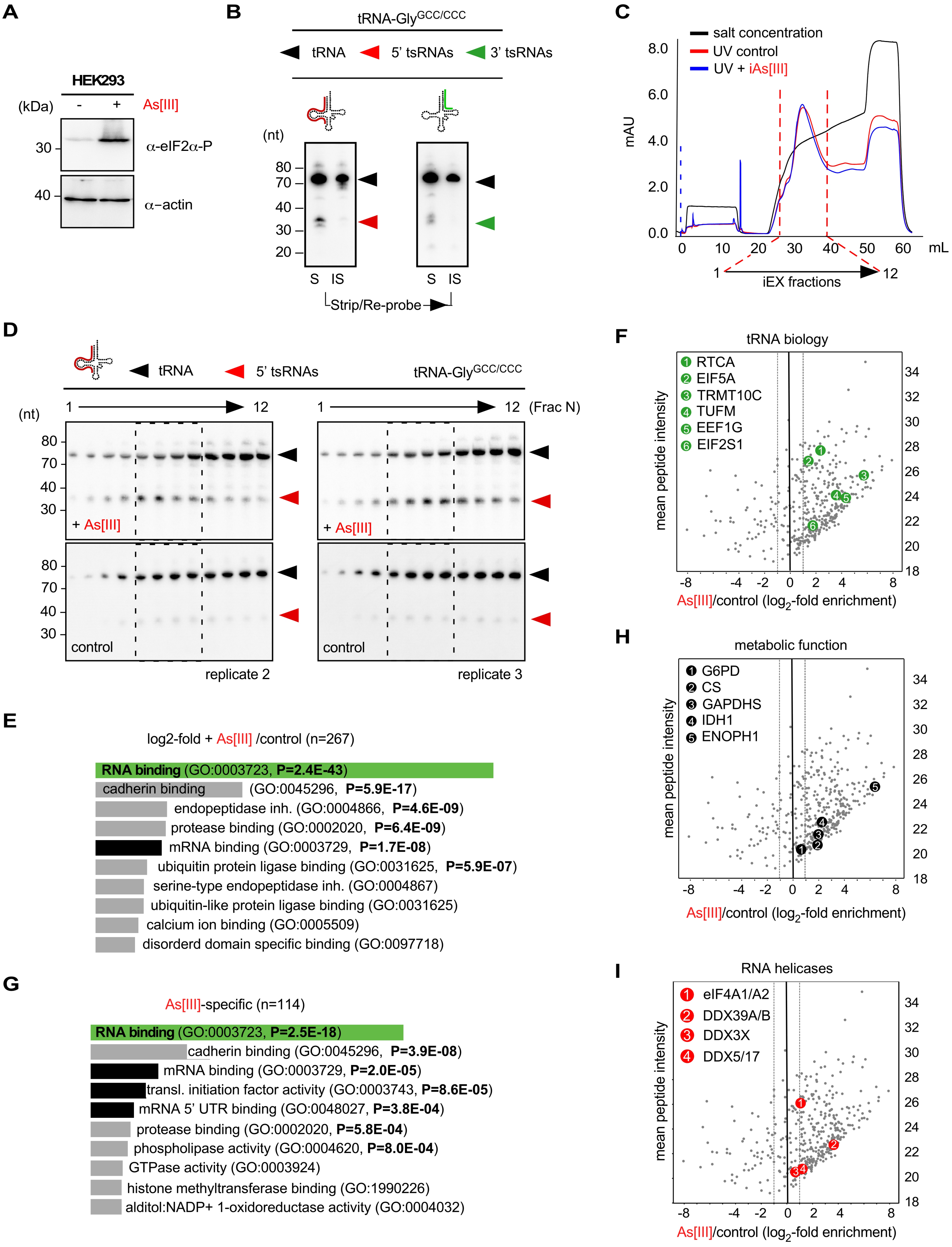
A
HEK293
(kDa)
- +
As[III]
30
α-eIF2α-P
40
α–actin
B
tRNA-Gly[GCC/CCC]
tRNA
5' tsRNAs
3' tsRNAs
(nt)
80
70
40
30
20
S IS
S IS
Strip/Re-probe
C
salt concentration
UV control
UV + iAs[III]
mAU
8.0
6.0
4.0
2.0
0.0
0 10 20 30 40 50 60 mL
1
iEX fractions
12
D
tRNA
5' tsRNAs
tRNA-Gly[GCC/CCC]
1 12
1 12 (Frac N)
(nt)
80
70
40
30
+ As[III]
control
replicate 2
replicate 3
E
log2-fold + As[III] /control (n=267)
RNA binding (GO:0003723, P=2.4E-43)
cadherin binding (GO:0045296, P=5.9E-17)
endopeptidase inh. (GO:0004866, P=4.6E-09)
protease binding (GO:0002020, P=6.4E-09)
mRNA binding (GO:0003729, P=1.7E-08)
ubiquitin protein ligase binding (GO:0031625, P=5.9E-07)
serine-type endopeptidase inh. (GO:0004867)
ubiquitin-like protein ligase binding (GO:0031625)
calcium ion binding (GO:0005509)
disorderd domain specific binding (GO:0097718)
F
tRNA biology
1 RTCA
2 EIF5A
3 TRMT10C
4 TUFM
5 EEF1G
6 EIF2S1
mean peptide intensity
34 32 30 28 26 24 22 20 18
-8 -4 -2 0 2 4 8
As[III]/control (log2-fold enrichment)
G
As[III]-specific (n=114)
RNA binding (GO:0003723, P=2.5E-18)
cadherin binding (GO:0045296, P=3.9E-08)
mRNA binding (GO:0003729, P=2.0E-05)
transl. initiation factor activity (GO:0003743, P=8.6E-05)
mRNA 5' UTR binding (GO:0048027, P=3.8E-04)
protease binding (GO:0002020, P=5.8E-04)
phospholipase activity (GO:0004620, P=8.0E-04)
GTPase activity (GO:0003924)
histone methyltransferase binding (GO:1990226)
alditol:NADP+ 1-oxidoreductase activity (GO:0004032)
H
metabolic function
1 G6PD
2 CS
3 GAPDHS
4 IDH1
5 ENOPH1
mean peptide intensity
34 32 30 28 26 24 22 20 18
-8 -4 -2 0 2 4 8
As[III]/control (log2-fold enrichment)
I
RNA helicases
1 eIF4A1/A2
2 DDX39A/B
3 DDX3X
4 DDX5/17
mean peptide intensity
34 32 30 28 26 24 22 20 18
-8 -4 -2 0 2 4 8
As[III]/control (log2-fold enrichment)

Supplementary Figure 2. Protein identity of RNPs co-migrating with 5' tsRNAs

(**A**) Western blot analysis of total protein extracts from HEK293 cells for the phosphorylated form of eIF2α (serine-51 phospho) before and after As[III] exposure. Antibodies against β-actin were used as a loading control.

(**B**) NB of total RNA extracted from soluble and insoluble HEK293 protein lysate obtained from HEK293 cells after exposure to As[III] (0.5 mM) using sequential probing of the same membrane against the 5' and 3' ends of tRNA-Gly$^{GCC/CCC}$. Black arrowheads, mature tRNAs; red arrowhead, 5' tsRNAs; green arrowhead, 3' tsRNAs. S, soluble fraction; IS, insoluble fraction.

(**C**) Superimposed chromatograms representing the IEX elution patterns of RNPs originating from a representative control (blue line) or an As[III] exposure experiment (red line); mAU, milli- absorbance units. IEX fractions denoted by dashed lines (1-12) were probed for tRNA-Gly$^{GCC/CCC}$ using NB.

(**D**) NB on total RNA extracted from fractions (N=12) that were obtained from subjecting 5' tsRNA-containing SEC fractions from replicate experiments to IEX using a probe against tRNA-Gly$^{GCC/CCC}$. Description as in **Figure 2D**.

(**E**) Gene ontology molecular function analysis (performed in ENRICHR) for proteins enriched more than two-fold in RNPs obtained after As[III] exposure (n=267). P-values are indicated within parentheses following GO annotation with ID number.

(**F**) Detail from scatter plot depicted in Figure 2F. Proteins connected to tRNA biology are highlighted as numbered green dots.

(**G**) Gene ontology molecular function analysis (performed in ENRICHR) for proteins detected specifically after exposure to As[III] (n=114). P-values are indicated within parentheses following GO annotation with ID number.

(**H**) Detail from scatter plot depicted in **Figure 2F**. Proteins with molecular function "metabolic" are highlighted as numbered black dots.

(**I**) Detail from scatter plot depicted in **Figure 2F**. RNA helicases are highlighted as numbered red dots. Multiple annotations (of different candidate helicases) are due to ambiguous mapping of detected peptides to multiple protein identities.

Drino_Figure S3

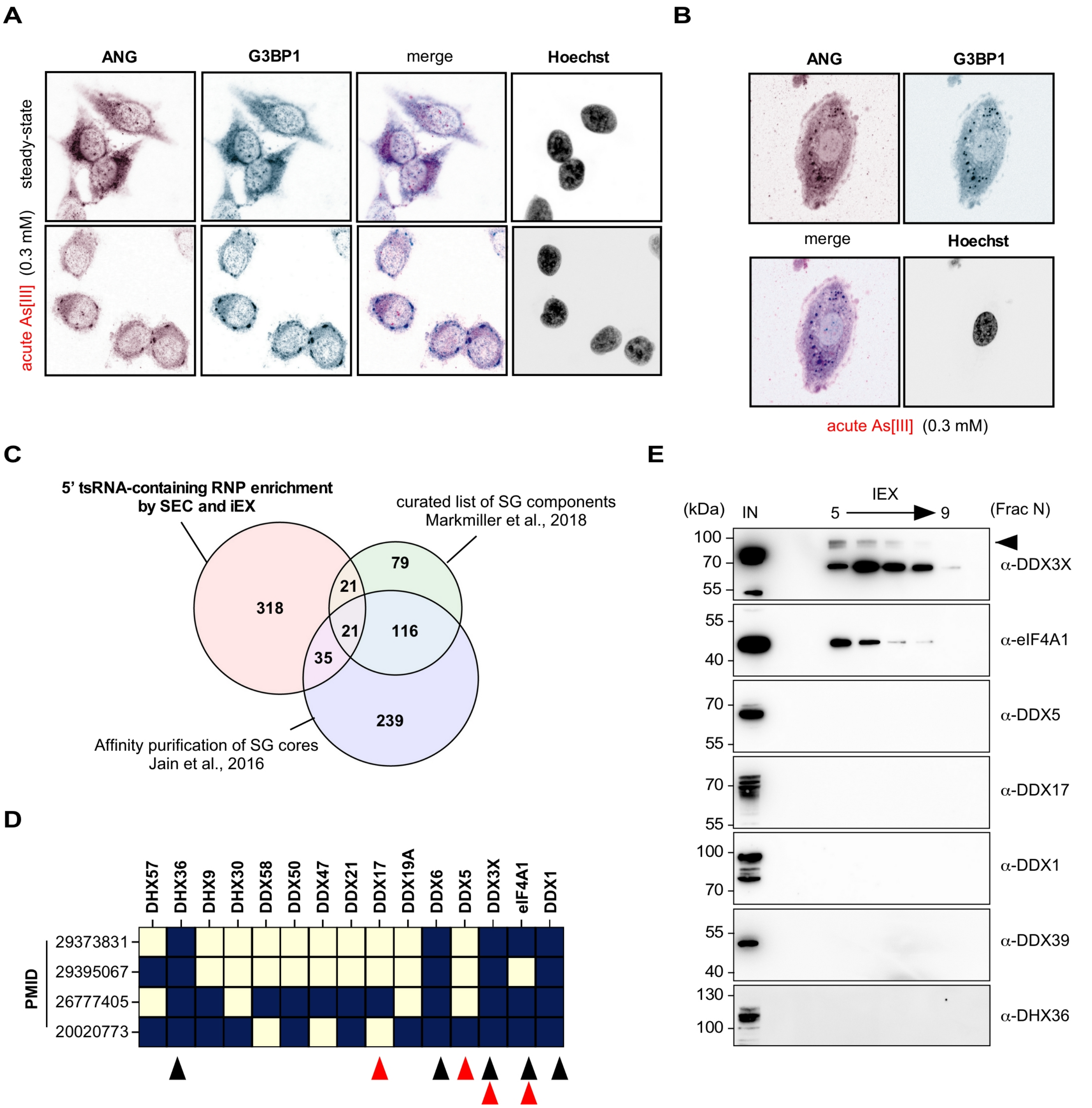

Supplementary Figure 3. SG association of ANG and 5' tsRNA-containing RNPs

(**A**) Indirect immunofluorescence images (false-coloured) of HeLa cells before and after acute exposure to As[III] (0.3 mM for one hour) using antibodies against human ANG (magenta) and G3BP1 (cyan).

(**B**) Indirect immunofluorescence images (false-coloured) of a U2OS cell after acute exposure to As[III] (0.3 mM for one hour) using antibodies against human ANG (magenta) and G3BP1 (cyan).

(**C**) Venn diagram showing the overlap of protein identities detected by biochemical fractionation of 5' tsRNA-containing RNPs (this work) with previously reported SG proteomes (94-97).

(**D**) Schematic compilation of RNA helicase identities that were reported in four publications to be connected to SG biology (PMIDs are referenced). Dark squares, in public record; pale yellow squares, no public record; black arrowheads, reported in all referenced reports; red arrowheads, co-migrating with 5' tsRNAs-containing RNPs (this work).

(**E**) Western blotting of IEX fractions exhibiting a positive 5' tsRNA-Gly$^{GCC/CCC}$/tRNA-Gly$^{GCC/CCC}$ signal ratio on NB (as in **Figure 2D** and Supplementary **Figure 2D**) for particular RNA helicases that were repeatedly associated with SG biology, and also identified in 5' tsRNAs-containing RNPs (this work). Input lane represents total HEK293 protein extract. IEX fraction numbers between 5 and 9 are depicted as an arrow. Black arrowhead denotes DDX3X migrating with a distinct mass up-shift, potentially revealing a fraction of DDX3X that was cross-linked to small RNAs such as tRNAs or tsRNAs (molecular mass of 23 kDa or 12 kDa, respectively).

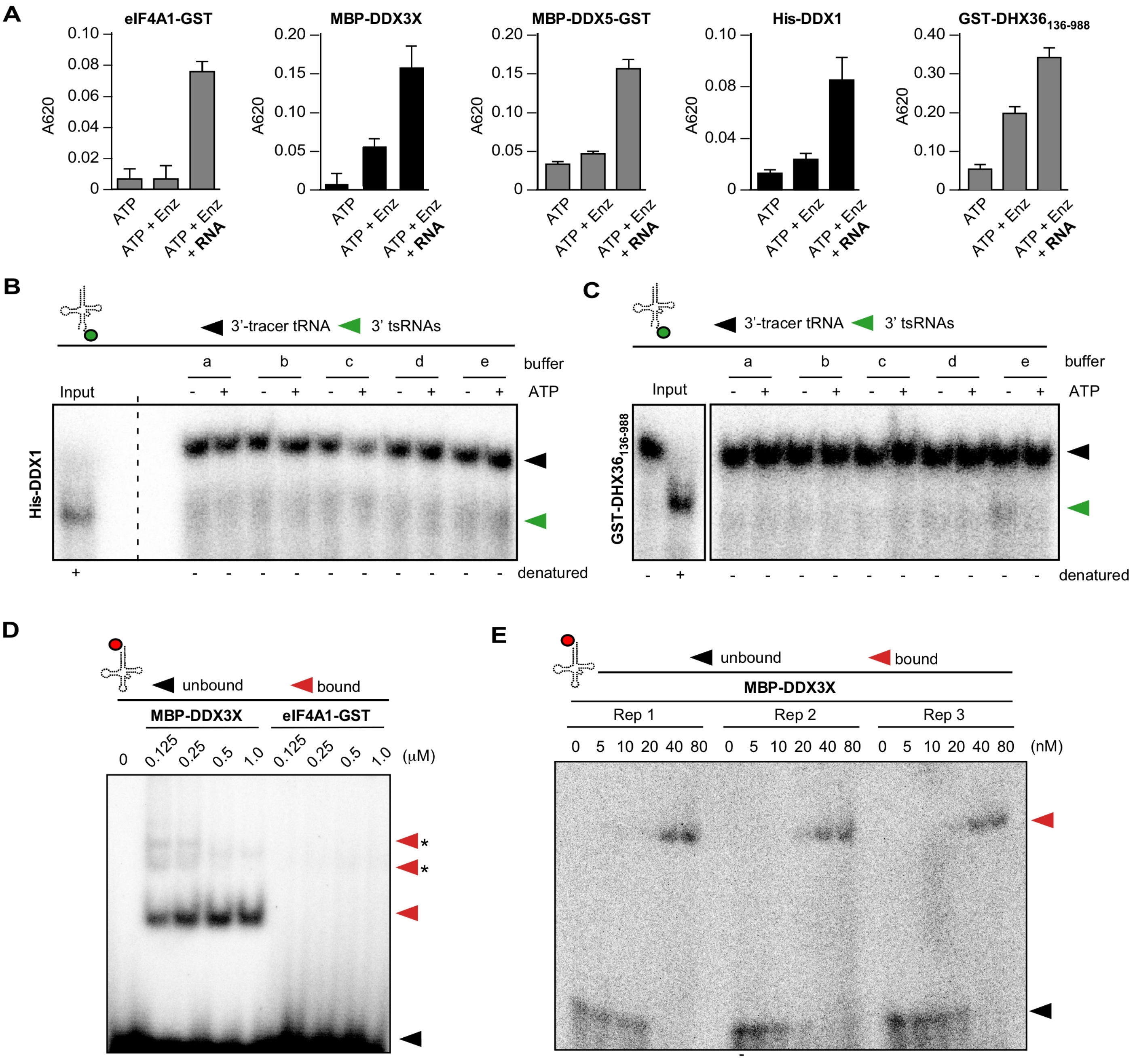
A
eIF4A1-GST
MBP-DDX3X
MBP-DDX5-GST
His-DDX1
GST-DHX36136-988
A620
0.10
0.08
0.06
0.04
0.02
0
0.20
0.15
0.10
0.05
0.40
0.30
0.20
ATP
ATP + Enz
ATP + Enz + RNA
B
3'-tracer tRNA
3' tsRNAs
a
b
c
d
e
buffer
Input
ATP
His-DDX1
denatured
C
GST-DHX36136-988
D
unbound
bound
MBP-DDX3X
eIF4A1-GST
0
0.125
0.25
0.5
1.0
(µM)
*
E
MBP-DDX3X
Rep 1
Rep 2
Rep 3
0 5 10 20 40 80
(nM)

Supplementary Figure 4. Binding and ATP activity assays identify recombinant DDX3X as interactor with tRNAs

(**A**) Colorimetric ATPase activity assays (fixed time-point) after combining fusion proteins (500 nM) with equimolar ATP/$MgCl_2$ (2 mM) and a single-stranded synthetic RNA oligo (1 µM) for 30 minutes. Individual bars represent the mean of technical triplicate experiments originating from three independent protein fractions after SEC. Error bars depict standard deviations.

(**B**) 3'-tracer tRNAs (8 nM final) were incubated with recombinant His-DDX1 dialysed into 5 different reaction buffers (a-e, for description, see **Supplemental Figure 1G**) in the presence or absence of 2 mM ATP. Reactions were separated by nPAGE and 3' tsRNA signals were collected as described above. To control for 3' tsRNA migration, 3'-tracer tRNAs were sampled after heat denaturation (Input). Black arrowhead, 3'-tracer tRNAs; green arrowhead, 3' tsRNAs.

(**C**) 3'-tracer tRNAs (8 nM final) were incubated with recombinant GST-$DHX36_{136\text{-}988}$ dialysed into 5 different reaction buffers (a-e, for description, see **Supplemental Figure 1G**) in the presence or absence of 2 mM ATP. Reactions were separated by nPAGE and 3' tsRNA signals were collected as described above. Black arrowhead, 3'-tracer tRNAs; green arrowhead, 3' tsRNAs.

(**D**) Representative EMSA after combining increasing molarities of MBP-DDX3X or eIF4A1-GST and 5' end-labelled tRNAs (30 nM final). UV-crosslinked RNPs were separated using nPAGE. Black arrowhead, unbound tRNAs; red arrowheads, DDX3X-tRNA complexes; asterisks indicate additional tRNA-derived signals, potentially revealing multimeric RNPs.

(**E**) Replicates of fixed time-point EMSA after combining increasing molarities of MBP-DDX3X ´and 5' end-labelled tRNA-$Lys^{UUU/CUU}$ (10 nM final) in the presence of equimolar AMP-PNP/$MgCl_2$ (2 mM). Black arrowhead, unbound tRNAs; red arrowhead, DDX3X-tRNA-$Lys^{UUU/CUU}$ complexes.

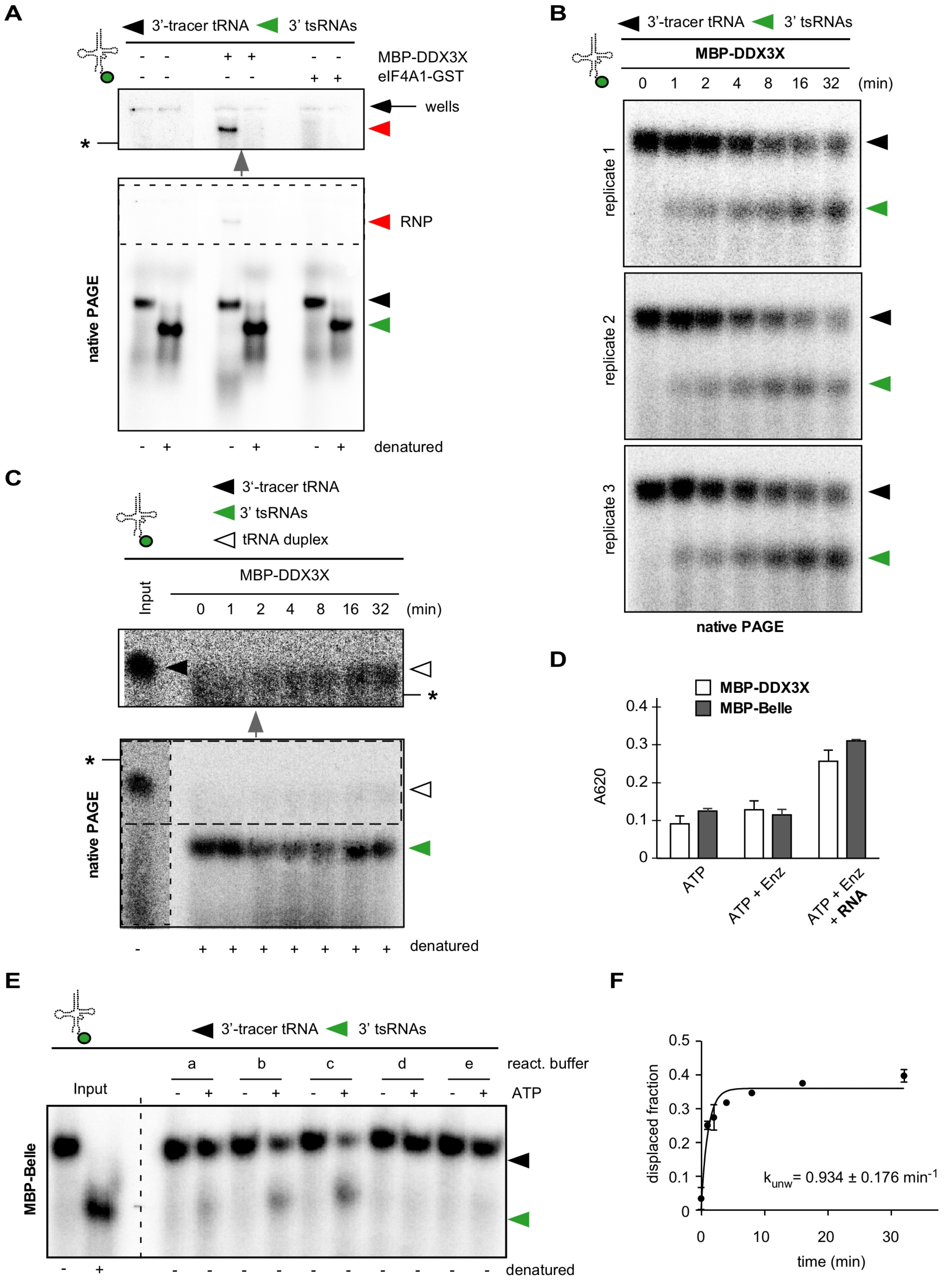
A
3'-tracer tRNA
3' tsRNAs
MBP-DDX3X
eIF4A1-GST
wells
RNP
native PAGE
denatured
B
3'-tracer tRNA
3' tsRNAs
MBP-DDX3X
0 1 2 4 8 16 32 (min)
replicate 1
replicate 2
replicate 3
native PAGE
C
3'-tracer tRNA
3' tsRNAs
tRNA duplex
MBP-DDX3X
Input
0 1 2 4 8 16 32 (min)
native PAGE
denatured
D
MBP-DDX3X
MBP-Belle
A620
0.4
0.3
0.2
0.1
0
ATP
ATP + Enz
ATP + Enz + RNA
E
3'-tracer tRNA
3' tsRNAs
Input
a
b
c
d
e
react. buffer
ATP
MBP-Belle
denatured
F
0.5
0.4
0.3
0.2
0.1
0
displaced fraction
k_{unw} = 0.934 ± 0.176 min^{-1}
0 10 20 30
time (min)

Supplementary Figure 5. Human DDX3X and its *Drosophila homologue* unwind 3'-tracer tRNAs

(**A**) Representative EMSAs after combining equal molarities of MBP-DDX3X and eIF4A1-GST (each 750 nM) and 3'-tracer tRNAs (10 nM final) in the presence of equimolar AMP-PNP/$MgCl_2$ (2 mM) for 30 minutes. UV-crosslinked RNPs were separated using nPAGE. Black arrowhead, 3'-tracer tRNAs; green arrowhead, 3' tsRNAs; dashed vertical box marked by asterisk, region digitally enhanced in upper panel; red arrowheads, RNPs containing 3'-tracer tRNAs and MBP-DDX3X.

(**B**) Replicate time-course RNA helicase activity assays (n=3) using MBP-DDX3X (750 nM) and 3'-tracer tRNAs (20 nM final) in the presence of equimolar ATP/$MgCl_2$ (2 mM). Aliquots were removed from reactions at indicated time points and separated using nPAGE. Black arrowheads, 3'-tracer tRNAs; green arrowheads, 3' tsRNAs.

(**C**) Strand-annealing assay using MBP-DDX3X (750 nM) on heat-denatured 3'-tracer tRNAs (10 nM final) as substrate without added ATP. Aliquots were removed from reactions at indicated time points and separated using nPAGE. Dashed vertical box marked by asterisk, region digitally enhanced in upper panel; Dashed horizontal box marked by asterisk, region digitally enhanced in upper panel; black arrowhead, 3'-tracer tRNAs; white arrowhead, expected size of re-annealed tRNA duplexes; green arrowhead, 3' tsRNAs.

(**D**) Colorimetric ATPase activity assays (fixed time-point) after combining MBP-belle (200 nM) at equimolar ATP/$MgCl_2$ (2 mM) concentrations and a single-stranded synthetic RNA oligo (1 µM) for 30 minutes. Individual bars represent the mean of technical triplicates originating from three independent protein fractions after SEC. Error bars depict standard deviations.

(**E**) 3'-tracer tRNAs (8 nM final) were incubated with recombinant MBP-belle dialysed into 5 different reaction buffers (a-e, see **Supplementary Figure 1G**) in the presence or absence of 2 mM ATP. Reactions were separated by nPAGE and 3' tsRNA signals were collected as described above. To control for 3'-tracer tRNA and 3' tsRNA migration, 3'-tracer tRNAs were sampled before and after heat denaturation (Input). Black arrowhead, 3'-tracer tRNAs; green arrowhead, 3' tsRNAs.

(**F**) Quantification of duplicate time-course RNA helicase activity assays using MBP-belle (500 nM) and 3'-tracer tRNAs (20 nM) produced from purified *D. melanogaster* tRNA in the presence of equimolar ATP/$MgCl_2$ to derive an unwinding constant. Line marks the fit of the mean values to the integrated first order rate equation, while error bars represent standard deviations (for calculation of values, see **Supplemental Table 4**).

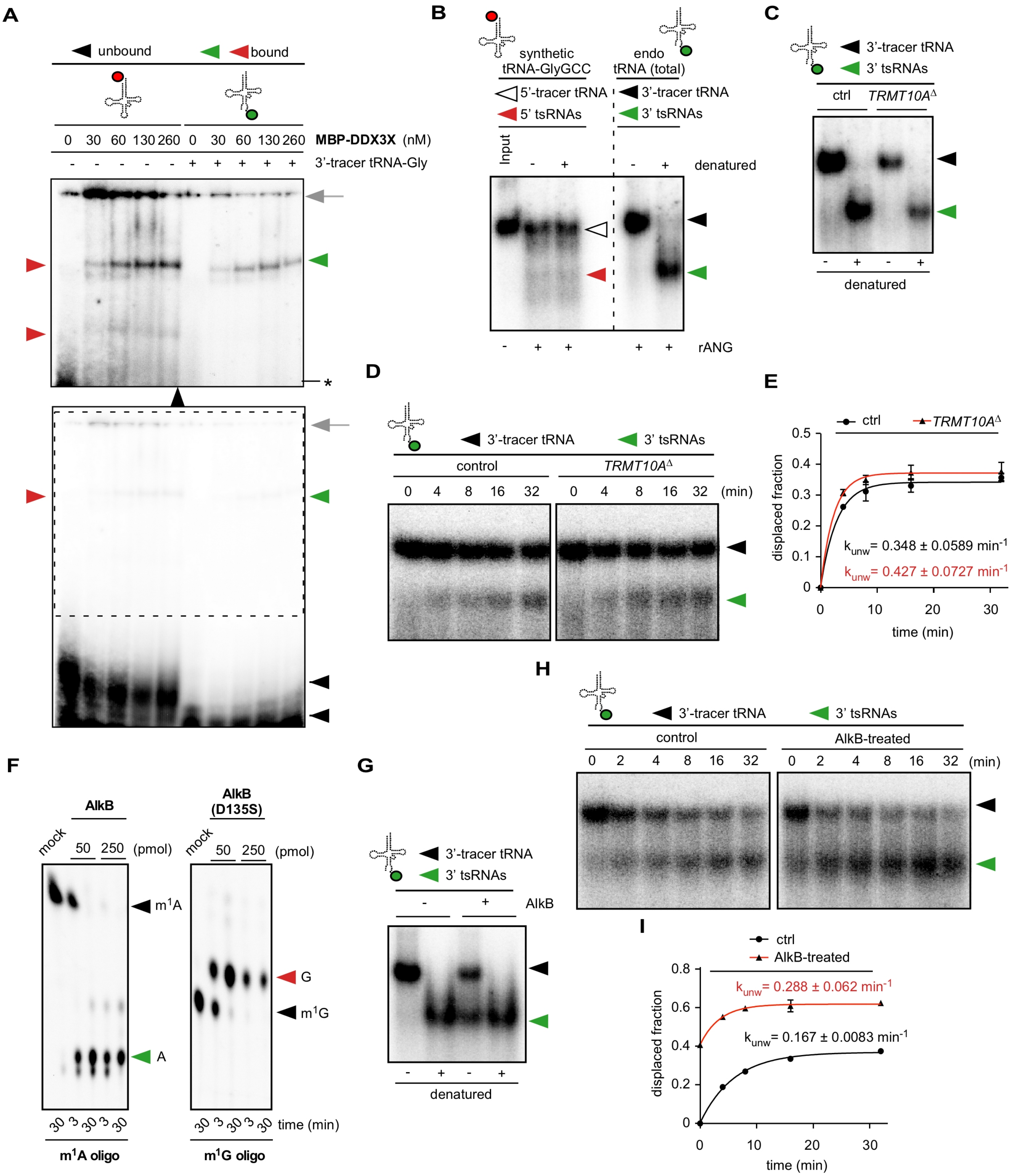
A
unbound
bound
0 30 60 130 260 0 30 60 130 260
MBP-DDX3X (nM)
3'-tracer tRNA-Gly
B
synthetic tRNA-GlyGCC
endo tRNA (total)
5'-tracer tRNA
5' tsRNAs
3'-tracer tRNA
3' tsRNAs
Input
denatured
rANG
C
3'-tracer tRNA
3' tsRNAs
ctrl
TRMT10AΔ
denatured
D
3'-tracer tRNA
3' tsRNAs
control
TRMT10AΔ
0 4 8 16 32 0 4 8 16 32 (min)
E
ctrl
TRMT10AΔ
displaced fraction
k_unw= 0.348 ± 0.0589 min-1
k_unw= 0.427 ± 0.0727 min-1
time (min)
F
AlkB
AlkB (D135S)
mock
50 250 (pmol)
m1A
G
m1G
A
30 3 30 3 30
time (min)
m1A oligo
m1G oligo
G
3'-tracer tRNA
3' tsRNAs
AlkB
denatured
H
3'-tracer tRNA
3' tsRNAs
control
AlkB-treated
0 2 4 8 16 32 0 2 4 8 16 32 (min)
I
ctrl
AlkB-treated
k_unw= 0.288 ± 0.062 min-1
k_unw= 0.167 ± 0.0083 min-1
displaced fraction
time (min)

Supplemental Figure 6. Specific tRNA modifications impact 3'-tracer tRNA structure but not DDX3X activity

(**A**) Representative EMSA after combining MBP-DDX3X (750 nM) with 5' end-labelled tRNA-Gly$^{GCC/CCC}$ or 3'-tracer tRNA-Gly$^{GCC/CCC}$ (20 nM final) in the presence of equimolar AMP-PNP/$MgCl_2$ (2 mM). Black arrowhead, unbound tRNAs or 3'-tsRNA, respectively; green arrowheads, 3'-tracer tRNA-Gly$^{GCC/CCC}$ bound by DDX3; red arrowheads, 5' end-labelled tRNA-Gly$^{GCC/CCC}$ bound by MBP-DDX3; grey arrow depicts material in wells. Upper panel (marked by asterisk) shows a digitally enhanced region of the gel region (lower dashed horizontal box).

(**B**) Testing of 5'-tracer tRNAs produced from synthetic tRNA-Gly^{GCC} for structural integrity using nPAGE. 5'-tracer tRNA integrity was sampled before and after heat denaturation. As controls, 3'-tracer tRNAs produced from HeLa cells were tested in parallel. White arrowhead, 5'-tracer tRNA; red arrowhead, position of 5' tsRNAs; black arrowhead, 3'-tracer tRNAs; green arrowhead, 3' tsRNAs.

(**C**) Testing of 3'-tracer tRNAs produced from HAP1 cells containing a wildtype TRMT10A gene (ctrl) or harbouring a null mutation in TRMT10A (*TRMT10A∆)* for structural integrity using nPAGE. 3'-tracer tRNA integrity was sampled before and after heat denaturation. Black arrowhead, 3'-tracer tRNAs; green arrowhead, 3' tsRNAs.

(**D**) Representative time-course RNA helicase activity assay using MBP-DDX3X (750 nM) and 3'-tracer tRNAs (20 nM final) produced from HAP1 cells (control and *TRMT10A∆)* in the presence of equimolar ATP/$MgCl_2$ (2 mM). Aliquots were removed from reactions at indicated time points and separated using nPAGE. Black arrowhead, 3'-tracer tRNAs; green arrowhead, 3' tsRNAs.

(**E**) Quantification of duplicate time-course RNA helicase activity assays (for calculation of values, see **Supplemental Table 4**) using MBP-DDX3X (750 nM) and 3'-tracer tRNAs (10 nM final) produced from HAP1 cells (described in **C**, **D**) in the presence of equimolar ATP/$MgCl_2$ (2 mM) to derive unwinding constants. Line marks the fit of the mean values to the integrated first-order rate equation, while error bars represent standard deviations.

(**F**) Thin-Layer-Chromatography (TLC) for activity testing of purified recombinant AlkB forms (50 and 250 pmol of AlkB and $AlkB_{D135S}$) on ^{32}P-labelled RNA oligonucleotides harbouring a single 5' modification (m^1A and m^1G, respectively) for 3 or 30 minutes at 25°C. After demethylation treatment, the oligoes were hydrolysed with P1 and resolved by TLC. Black arrowheads, 5' monophosphate m^1A and m^1G, respectively; green arrowhead, 5' monophosphate adenine; red arrowhead, 5' monophosphate guanosine.

(**G**) Testing of 3'-tracer tRNAs produced from HeLa cells treated with recombinant AlkB forms for structural integrity using nPAGE. 3'-tracer tRNA integrity was sampled before and after heat denaturation. Black arrowhead, 3'-tracer tRNAs; green arrowhead, 3' tsRNAs.
(**H**) Representative time-course RNA helicase activity assay using MBP-DDX3X (750 nM) and 3'-tracer tRNAs (20 nM final) produced from HeLa cells treated with recombinant AlkB forms in the presence of equimolar ATP/$MgCl_2$ (2 mM). Aliquots were removed from reactions at indicated time points and separated using nPAGE. Black arrowhead, 3'-tracer tRNAs; green arrowhead, 3' tsRNAs.
(**I**) Quantification of duplicate time-course RNA helicase activity assays (for calculation of values, see **Supplemental Table 4**) using MBP-DDX3X (750 nM) and 3'-tracer tRNAs (10 nM final) produced from HeLa cells treated with recombinant AlkB forms (described in **G**) in the presence of equimolar ATP/$MgCl_2$ (2 mM) to derive unwinding constants. Line marks the fit of the mean values to the integrated first-order rate equation, while error bars represent standard deviations.

Drino_Figure S7

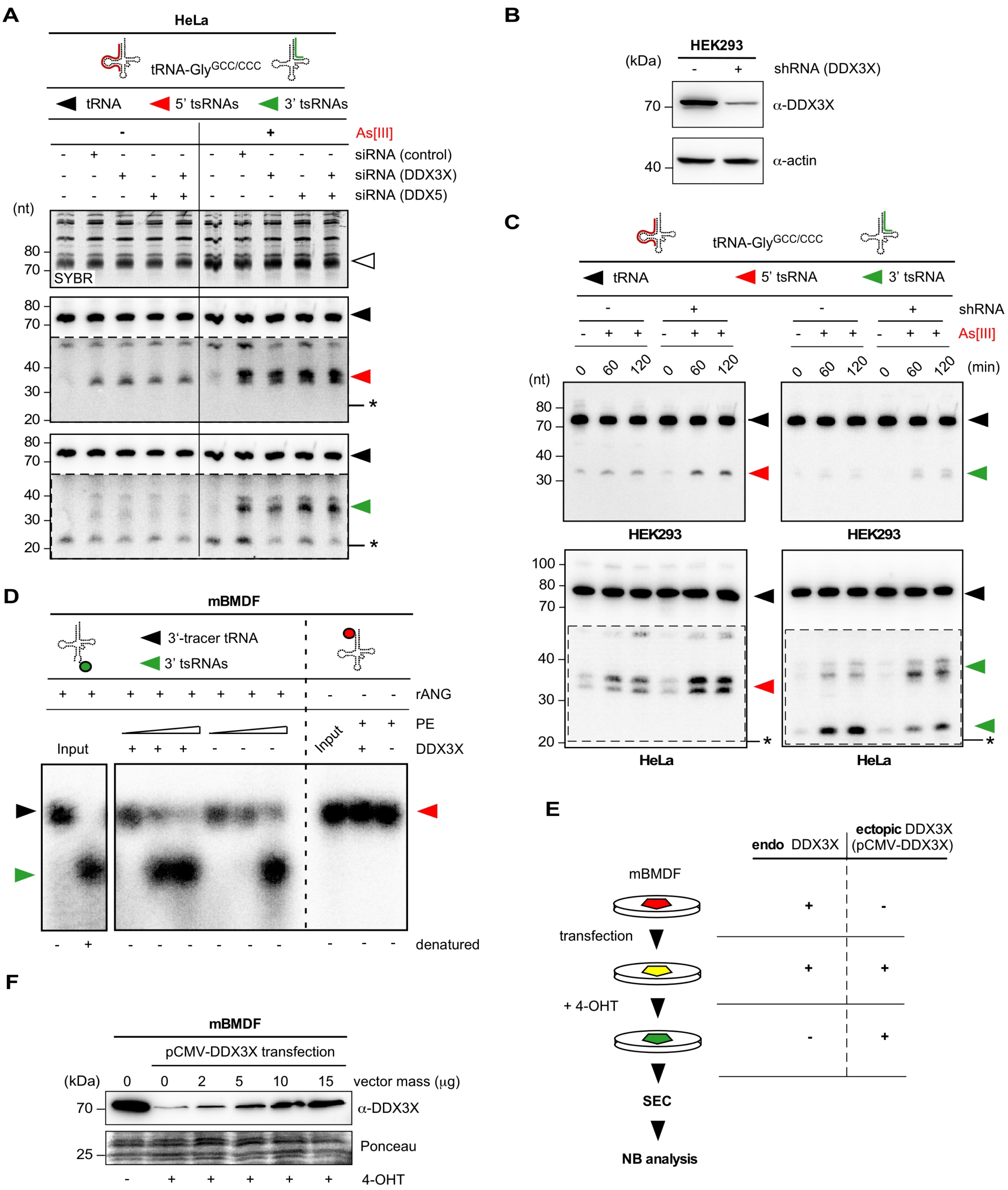

Supplementary Figure 7. Specific tRNAs and stress-induced tsRNAs levels are unaffected after depletion of DDX3X

(**A**) NB using probes against the 5' and 3' portions of tRNA-Gly$^{GCC/CCC}$ on total RNA purified from HeLa cells 48 hours after transfection with siRNA pools (non-targeting as controls versus targeting each RNA helicase alone or in combination) and before and after As[III] exposure (0.5 mM for one hour). Top panels, SYBR staining; bottom panels, NB; black arrowheads, mature tRNAs; red arrowhead, 5' tsRNAs; green arrowhead, 3' tsRNAs. Lower panels (marked by asterisk) show a digitally enhanced region of the membrane region (dashed horizontal box).

(**B**) Western blotting for DDX3X in total protein extract obtained from HEK293 cells after shRNA-mediated knock-down (48 hours) of DDX3X. β-actin was used as loading control.

(**C**) NB using probes against the 5' and 3' portions of tRNA-Gly^{GCC} on total RNA purified from HEK293 and HeLa cells after shRNA-mediated knock-down of DDX3X (72 hours) before and after exposure to iAs (0.5 mM for 60 or 120 minutes). Black arrowheads, mature tRNAs; red arrowheads, 5' tsRNAs; green arrowheads, 3' tsRNAs.

(**D**) Fixed-time point RNA helicase activity assays using increasing concentrations (2.5, 5 and 10 µg) of cytoplasmic protein extract obtained from BMDF (+/- DDX3X) and 3'-tracer tRNAs (8 nM final) in the presence of equimolar ATP/$MgCl_2$ (2 mM) are depicted left of the dashed vertical line. Panel to the right of dashed vertical line depicts incubation of cytoplasmic protein extract (5 µg) obtained from BMDF (+/- DDX3X) with 5' end-labelled tRNAs (8 nM final) in the presence of equimolar ATP/$MgCl_2$ (2 mM) to control for tRNA degradation. 3'-tracer tRNAs that were heat-denatured (Input) are shown in an individual panel on the left. Reactions were separated by nPAGE and ^{32}P-signals were collected as described above. Black arrowhead, 3'-tracer tRNAs; red arrowhead, 5' end-labelled tRNAs; green arrowhead: 3' tsRNAs.

(**E**) Schematic representation of ectopic over-expression of CMV-driven murine DDX3X in DDX3X-deficient BMDF. BMDF (red, DDX3X endo +) were transfected with a plasmid encoding murine DDX3X cDNA (yellow, DDX3X endo +, ectopic +), followed by 4-OHT-mediated deletion of endogenous DDX3X (green, DDX3X endo -, ectopic +). Cells over-expressing ectopic DDX3X were used for downstream biochemical fractionation and NB experiments.

(**F**) Western blotting for DDX3X performed on total protein extract derived from BMDF 96 hours after transfection with increasing amounts of plasmid encoding DDX3X cDNA and before and after 4-OHT mediated deletion of the endogenous DDX3X genetic locus for 72 hours. Ponceau staining of total protein was used as loading control.
